# Detection of Adulterants in Powdered Foods Using Near-Infrared Spectroscopy and Chemometrics: Recent Advances, Challenges, and Future Perspectives

**DOI:** 10.3390/foods14183195

**Published:** 2025-09-13

**Authors:** William Vera, Rebeca Salvador-Reyes, Grimaldo Quispe-Santivañez, Guillermo Kemper

**Affiliations:** 1 Programa de Doctorado en Ingeniería Agroindustrial Mención Transformación Avanzada de Granos y Tubérculos Andinos, Universidad Nacional del Santa, Nuevo Chimbote 02711, Ancash, Peru; 2025818010@uns.edu.pe; 2 Facultad de Ingeniería, Universidad Tecnológica del Perú, Lima 150101, Lima, Peru; rsalvador@utp.edu.pe; 3 Escuela Profesional de Ingeniería Agroindustrial, Facultad de Ingeniería, Universidad Nacional Autónoma Altoandina de Tarma, Acobamba 120701, Tarma, Peru; gquispe@unaat.edu.pe; 4 Faculty of Engineering, School of Electronic Engineering, Universidad Peruana de Ciencias Aplicadas, Av. Prolongación Primavera 2390, Monterrico, Santiago de Surco, Lima 15023, Lima, Peru

**Keywords:** food fraud, spectroscopy, non-destructive, machine learning, deep learning

## Abstract

Powdered foods are matrices transformed into fine, loose solid particles through dehydration and/or milling, which enhances stability, storage, and transport. Due to their high commercial value and susceptibility to fraudulent practices, detecting adulterants in powdered foods is essential for ensuring food safety and protecting consumer health and the economy. Food fraud in powdered products, such as spices, cereals, dairy-based powders, and dietary supplements, poses an increasing risk to public health and consumer trust. These products were selected as representative matrices due to their high nutritional and economic relevance, which also makes them more susceptible to adulteration and hidden potential health risks from hidden contaminants. Recent studies highlight the potential of spectroscopic techniques combined with chemometrics as rapid, non-destructive, and cost-effective tools for authentication. This narrative review synthesizes recent literature (2020–2025) on the application of near-infrared (NIR) spectroscopy combined with chemometric techniques for adulterant detection in powdered foods. Advances in spectral preprocessing, variable selection, classification, and regression models are discussed alongside the most common adulterants and their nutritional and toxicological implications. Furthermore, the applicability of portable versus benchtop NIR devices is compared. The main contribution of this review lies in critically analyzing methodological frameworks, mapping current gaps, and identifying emerging trends, such as digital integration, self-adaptive chemometric models, and real-time on-site authentication, positioning NIR spectroscopy as a promising tool for food authentication and quality control.

## 1. Introduction

Food fraud poses a global threat to safety. According to the latest FAO report (2023), this phenomenon results in estimated annual economic losses of $40 billion and affects approximately 16,000 tons of food and beverages [1,2]. The magnitude of this issue not only undermines consumer trust but also compromises nutritional integrity and exposes populations to critical health risks [3,4,5], particularly in powdered foods—such as dairy products, spices, and flours—whose physical structure facilitates fraudulent practices [6,7]. This global problem transcends economic concerns and constitutes a major public health challenge [8].

Within this context, adulteration manifests in three main dimensions: intentional, accidental, and falsified [9,10,11]. Intentional cases include strategies such as substituting premium ingredients with by-products [12], for example, the use of ground walnut, peanut, and pecan shells in cinnamon [13], or the addition of low-cost compounds, such as starches in protein supplements [6,14]. However, adulteration is not always deliberate: failures in good production or manufacturing practices can lead to contamination with heavy metals in ground spices [15] or pesticide residues in fruits and vegetables [16,17], phenomena that, although unintentional, are equally hazardous [18]. Likewise, falsification involves misleading information, such as incorrect labeling or manipulation of geographical origin, as seen in gourmet coffee adulterated with lower-quality varieties [19]. The detection of synthetic pesticides in products labeled as “organic” is a notable example [16,20], illustrating how fraud continues to evolve to circumvent regulations.

These fraudulent practices can have direct consequences on human health. Risks range from immediate allergic reactions caused by hidden allergens [21,22,23,24] to chronic effects, such as neurotoxicity associated with lead in adulterated spices [25]. Historical cases, such as the melamine-contaminated powdered milk scandal reported in 2008—which affected 300,000 infants—demonstrate the catastrophic potential of such practices [26]. Epidemiological studies have linked unauthorized additives in protein supplements to progressive liver damage [27], reinforcing the need for detection systems capable of identifying fraud in powdered foods.

Advanced methods, such as high-performance liquid chromatography (HPLC), used exclusively at the laboratory level, or polymerase chain reaction (PCR), are effective for food authentication [28]. However, these analytical methods face critical limitations in this context. Although accurate, they require extended analysis times, involve high costs, and are destructive to the sample, which restricts their applicability in continuous monitoring [29,30]. Moreover, techniques such as PCR rely on specific reagents, rendering them ineffective against unknown adulterants, while mass spectrometry demands highly specialized personnel, limiting its industrial adoption [31]. These technical and operational barriers limit the availability of such methods in the food industry.

Several reviews have addressed the role of advanced analytical approaches in food authentication. For instance, Shi et al. [32] provided a broad overview of spectroscopic and chemometric techniques applied to diverse food products without focusing on the specific challenges of powdered matrices. Similarly, Usman et al. [33] analyzed both conventional and modern analytical strategies, including NIR spectroscopy, but did not provide a comparison of chemometric models in powdered foods. These contributions highlight the potential of NIR-based approaches but also reveal a gap in specialized reviews centered on powdered products.

NIR spectroscopy emerges as a disruptive solution in this scenario. Based on molecular vibrations of C–H, N–H, and O–H bonds [34,35], this technique enables the rapid and accurate identification of adulterants in powdered foods while preserving the sample [32,36,37]. Its integration with advanced chemometrics—such as regression models or neural networks—has raised detection accuracy to over 90% in cases of food fraud involving powdered dairy products [36,38], spices [13,39,40], cereals [24,41], coffee [23], cocoa [42,43,44], and maca [45,46], among others. Furthermore, portable devices (900–1700 nm) enable on-site analysis, democratizing access to cutting-edge technology, and reducing logistical costs [37]. The combination of NIR and data analysis is not merely a tool but a key pillar in the global food supply chain [47].

Within this framework, the present review examines recent advances (2020–2025) in the application of NIR spectroscopy coupled with chemometrics, assessing its potential as an effective tool for detecting food fraud in powdered products, which are particularly vulnerable to such malpractice. Spectral strategies for common adulterants, chemometric algorithms (PCA, SVM, deep learning), and emerging trends are evaluated through an analysis of Scopus-indexed studies. The objective goes beyond a purely technological overview: this work proposes an integrated framework that prioritizes industrial scalability, analytical sensitivity, and alignment with the Sustainable Development Goals (SDGs 2 and 3).

## 2. General Principles of NIR Spectroscopy

**Figure 1:**
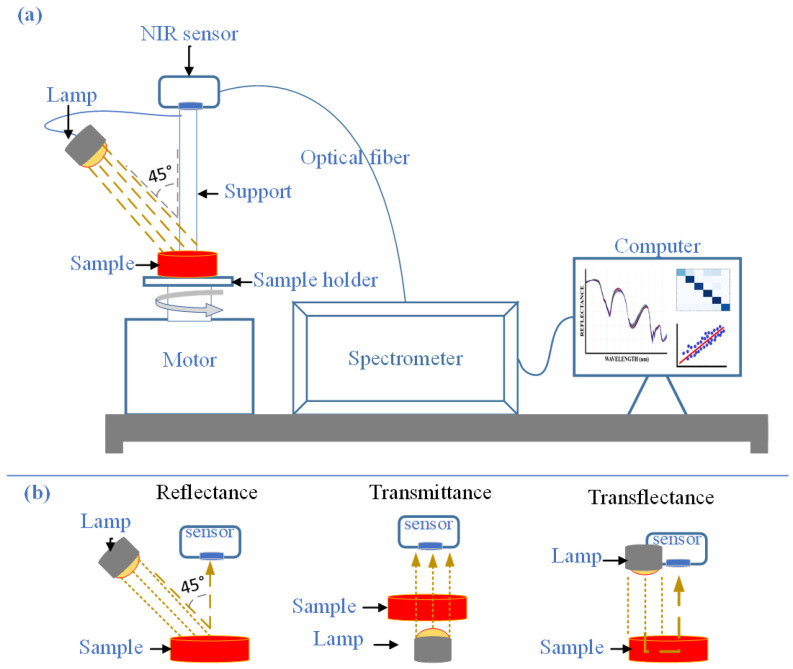
NIR Spectroscopy System: (**a**) Components of the NIR spectroscopy system; (**b**) Diagram of spectral acquisition modes. Adapted from Chikri et al. [40].

Near-infrared (NIR) spectroscopy is a technique based on the interaction of light with matter within the spectral range of 700–2500 nm. This range encompasses overtones and combination bands of molecular vibrations associated with C–H, N–H, and O–H bonds, which are typical of organic compounds. Light absorption occurs when the radiation energy matches the molecular vibration frequency, resulting in molecular excitation and generating a characteristic spectral signal [35,48]. As shown in Figure 1, a typical NIR system consists of a radiation source, a sample cell, and a detector and enables spectral acquisition through three main modes: reflectance, transmittance, and transflectance. Diffuse reflectance is the most widely used mode for powdered food matrices, such as coffee [23], cereals [22,24], dairy products [38,49], and powdered spices [39,42]. In contrast, transmittance is preferred for liquids or thin films, whereas transflectance is applied in specialized configurations that combine both phenomena [50,51,52,53].

Overtone and combination transitions are responsible for absorption in the NIR region, occurring primarily in hydrogen, carbon, and oxygen bonds. These vibrations, which are less intense than those in the mid-infrared (MIR) region, do not alter the sample, providing a significant advantage to the technique [54]. The process is modeled using the Beer–Lambert law, where the absorbance is proportional to both the concentration and the optical path length through the sample [50,55].

NIR spectroscopy is nondestructive, rapid, and does not require chemical reagents, making it both cost-effective and sustainable [35,51]. Its ability to perform simultaneous multicomponent analysis is particularly valuable in the field of food studies [52,53]. However, it has limitations, such as low sensitivity for components present at concentrations below 1% and the need for precise calibration models [56,57]. Additionally, it is affected by environmental factors [58,59] and variability between instruments [56,60].

## 3. Chemometrics Coupled with NIR for Fraud Detection

**Figure 2:**
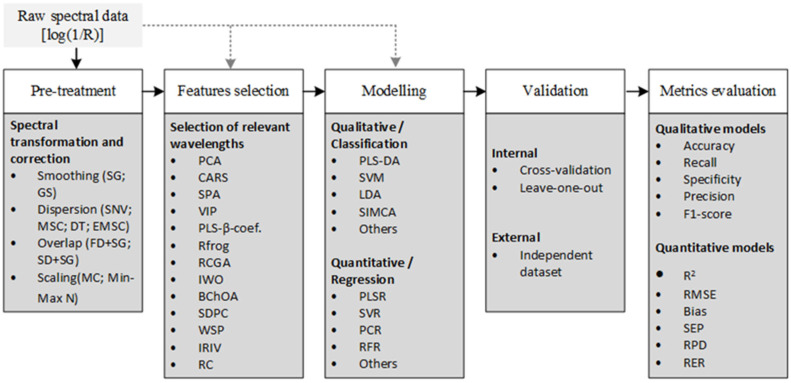
General workflow for chemometrics coupled with NIR spectroscopy in food fraud detection.

The development of robust chemometric models for detecting adulteration in powdered foods fundamentally depends on the following stages: spectral preprocessing, selection of relevant features, classification or regression model, validation, and evaluation of metrics [61,62]. The main strategies used in the reviewed studies are described below. Figure 2 illustrates the general workflow from raw spectral data acquisition to model evaluation to provide a comprehensive overview of this process.

### 3.1. Spectral Preprocessing Techniques

NIR spectra of powdered foods are influenced by factors such as moisture and particle size or geometry, which cause baseline shifts (additive effects) and slope changes due to light scattering (multiplicative effects). Sample surface irregularities and measurement parameters, including probe–sample distance, measurement angle, or packaging, may also cause additional noise [22,23,63]. To minimize these issues, the experimental design should include controlling the moisture content [37,43], standardizing the particle size [64,65,66], ensuring the surface uniformity [14,67], and harmonizing the measurement parameters [40,68].

In addition, preprocessing techniques are essential to correct residual effects. A wide range of methods—summarized in Table 1—are available, including baseline correction, peak resolution, scattering compensation, normalization, and scaling [23,63,69,70]. These techniques can be applied individually or in combination, depending on the spectral distortion complexity. For instance, MSC or SNV are sufficient to address scattering effects [63,67,70], whereas more complex interferences may require additional approaches, such as Savitzky–Golay smoothing, mean-centering, or autoscaling, which in many cases significantly enhance model performance [23,24,69,71,72].

This is particularly relevant in the acquisition of NIR spectra using a non-invasive approach, particularly through low-density polyethylene (LDPE) bags, which can cause optical scattering, additional absorption from the packaging, and variations in light path length, all of which affect spectral quality—especially in the 1800–2350 nm region [68,73,74]. To minimize these interferences, calibrating the reference spectra using the same packaging material, consistently using the same type of bag in all measurements, and properly homogenizing the product inside is recommended to ensure a stable surface. In such cases, spectral preprocessing techniques are essential. Commonly applied methods include the Savitzky–Golay method, derivatives, detrending, MSC, and SNV [45,64]. According to Lukacs et al. [68], SNV was used to correct multiplicative scatter effects, whereas Savitzky–Golay with first- or second-order derivatives smoothed the spectra and enhanced relevant signals over background noise. Empirically selected combinations, such as SG + SNV or SG + MSC, significantly improved model performance. These preprocessing steps were crucial for stabilizing the spectra acquired through plastic. Furthermore, LDPE introduced distinct absorption bands—particularly at 1220, 1800, and 2350 nm—that overlapped with phenolic compound bands, causing spectral distortions corrected through SNV, detrending, and derivatives [73].

SG has been widely adopted for spectral smoothing, reducing high-frequency noise while preserving the absorption band morphology [64,77,93]. Its combination with spectral derivatives enhances its ability to highlight subtle differences, facilitating the separation of samples with similar chemical compositions [43,90].

For example, Rukundo et al. [74] applied SG with a first-order derivative (second-degree polynomial, 61-point window) as a preprocessing step for a PCA-SIMCA model, achieving a correct classification rate of 97.4% for metanil yellow-adulterated samples. Similarly, Lukacs et al. [68] combined derivatives with SG and developed PLSR and SIMCA models with 100% sensitivity and specificity for melamine and urea detection in powdered milk. Chen et al. [66] used PLSR to integrate SG with SNV to improve the prediction of 6-gingerol in powdered ginger, obtaining an R^2^p of 0.9497 and an RPD of 4.23. Coqueiro et al. [75] applied SG to adulterated corn flour and reported a validation R^2^ of 0.9949 in an optimized PLS model. These results confirm that the use of SG, particularly in combination with derivatives or normalization techniques, substantially contributes to spectral stability and enhances the sensitivity of chemometric models.

SNV is one of the most commonly used techniques to correct variations caused by light scattering or differences in the physical presentation of samples. Lanjewar et al. [37] and Amsaraj et al. [81] applied RF and PLS models to adulterated turmeric and demonstrated that SNV enabled R^2^ values above 0.97 and RMSEP as low as 0.1696. Additionally, Chen et al. [66] reported that its combined application with SG improved the performance of the PLS model for ginger, achieving an RPD of 4.23.

MSC is effective for samples with high physical heterogeneity. In the PLS models developed by Chikri et al. [40] and Casarin et al. [22], the application of MSC resulted in prediction R^2^ values exceeding 97% and errors below 0.12% in protein and ash content quantification. Its combination with MC was also useful in improving multiclass discrimination through PLS-DA and SIMCA models, as demonstrated by Yegon et al. [72] and Lukacs et al. [68].

The extended version, EMSC, incorporates both linear and nonlinear components, providing a more accurate correction of physical variations. In the study by Ting et al. [49], EMSC was applied as the final step in a preprocessing sequence that included Cut, Gaussian smoothing, and normalization, leading to an improvement in the accuracy of a logistic-PCA model for detecting adulterated milk from 90% to 100%. In hierarchical SIMCA and quantitative PLSR models for melamine and urea detection, EMSC reduced RMSEP and increased sensitivity to over 97%.

First and Second Derivatives: Spectral derivatives are essential for baseline correction, resolving overlapping peaks, and enhancing spectral differences. In particular, the FD has proven useful in both classification and quantification models. Tao et al. [41] achieved 93.83% accuracy in a multiclass PLS-DA model by applying it in combination with SG (7-point window). Bala et al. [86], using a (1,4,4,1) configuration, reached an R^2^ of 0.999 and an RPD greater than 16 in MPLSR models applied to adulterated chickpea flour.

SD provided higher resolution for overlapping signals. Sadeghi et al. [39] integrated SD with SNV and detrending in adulterated turmeric, allowing a CNN model to achieve an R^2^ of 0.848 and an MAE of 3.15%. Casarin et al. [22] reported an R^2^p of 97.44% for lipid prediction in teff flour, whereas Ndlovu et al. [94] obtained an RPD of 6.23 for resistant starch quantification using PLS with second derivative preprocessing.

DT corrects non-informative systematic trends—such as baseline curvature—by fitting and subtracting a polynomial function. In the PLS and MPLSR models, DT significantly improved spectral linearity and reduced structural noise. Bala et al. [86] and Ndlovu et al. [90] reported R^2^ values up to 0.999 and SEP < 2.6% in chickpea flour and resistant starch, respectively. The SNV–DT combination applied in SVR models for buckwheat [83] achieved an R^2^p of 0.9987 and an RMSEP as low as 0.0002.

MC eliminates systematic bias across spectral variables, facilitating multivariate analysis. Its application, particularly in combination with 1D or MSC, has proven to be effective in PLS-DA models. Yegon et al. [72] achieved an R^2^p of 0.98 and an RMSEP of 2.74% in rice flour, while De Carvalho et al. [46] reported perfect sensitivity and specificity (1.000) for detecting adulteration in maca.

To optimize spectral relevance and reduce noise, complementary techniques, such as cut and Gaussian smoothing, have been employed. In adulterated powdered milk, Ting et al. [49] applied spectral trimming (980.943–1621.240 nm) followed by Gaussian smoothing, which enabled the PCA model to correctly classify 100% of the samples, compared to only 90% with untreated data.

Finally, Min–Max normalization has proven to be highly effective in contexts where spectral comparability is required without altering signal shapes. Kar et al. [65] identified this technique as the most effective among the seven evaluated methods, integrating it into a PLSR model for adulterated turmeric with Sudan I. The model achieved an RMSECV of 0.168 using only five latent variables, demonstrating high accuracy with low structural complexity.

Spectral preprocessing is a critical stage in NIR–chemometric analysis because it improves the quality of the data and directly influences the performance of models for adulteration detection [32]. The reviewed studies show that no single method is universally optimal; instead, the best results are usually obtained by combining approaches such as SG smoothing, first and second derivatives, SNV, MSC, EMSC, and detrending, which help reduce noise, correct baseline shifts, and minimize scattering effects in powdered food spectra. Importantly, some studies have evaluated derivatives separately and later applied SG filtering, which is why both appear as distinct techniques in Table 1, highlighting their individual and complementary contributions.

Preprocessing should not be considered a secondary step but rather an integral component of the analytical workflow. Although preprocessing generally enhances model accuracy and robustness, in some cases, raw spectra have yielded better predictions than preprocessed data [95]. This demonstrates that the specific characteristics of the food matrix and the type of adulteration under study must guide the choice of technique, ensuring reproducibility, reliability, and practical applicability in food authenticity and fraud detection.

### 3.2. Feature Selection Techniques

Dimensionality reduction and the selection of relevant variables are fundamental steps in chemometric modeling, particularly in NIR spectroscopy, where the number of wavelengths greatly exceeds the number of samples [22,63]. Inadequate variable selection may lead to overfitting, interpretability loss, and reduced generalization capacity [80,96]. In this context, various techniques—both linear and metaheuristic—have been applied to improve the predictive performance and robustness of the models. Table 2 summarizes the main methodologies employed in recent studies on powdered food authenticity, including Principal Component Analysis (PCA), Competitive Adaptive Reweighted Sampling (CARS), Successive Projections Algorithm (SPA), Variable Importance in Projection (VIP), and evolutionary algorithms, each with specific advantages and limitations depending on the type of matrix and adulterant evaluated.

PCA is an effective exploratory technique for reducing the dimensionality of spectral data, facilitating the visualization of latent patterns, and improving the computational efficiency of classification models. In studies such as that of Sadeghi et al. [39], its use prior to CNNs allowed compression of over 1000 wavelengths, reducing training time without compromising accuracy (R^2^ = 0.848). Similarly, Lanjewar et al. [37] employed PCA combined with SNV to detect starch-adulterated turmeric, achieving a 99.8% reduction in variables and high predictive performance (R^2^ = 0.999; F1 = 96%). Other studies have highlighted its usefulness for spectral interpretation. Ni et al. [95] applied PCA to associate specific bands with compounds such as chitin or lipids, whereas Boadu et al. [69] identified key spectral regions in coffee samples based on geographical origin. However, some studies have acknowledged its limitations. Chen et al. [66] reported that although PCA slightly improved performance over full-spectrum data, it outperformed RFrog in the detection of specific adulterants. Similarly, in multiclass models, Tao et al. [41] observed that reducing the dataset to three components was insufficient for robust discrimination.

CARS is one of the most effective techniques for wavelength selection in NIR spectral analysis applied to food adulteration detection. Its ability to significantly reduce the number of variables without compromising predictive performance has been validated in multiple studies. Chen et al. [100] reduced the number of wavelengths from 256 to between 5 and 44, depending on the model, improving SVR accuracy with an R^2^p of 0.7769 and an RPD of 2.32 in rice mixtures—surpassing even the full-spectrum model. Similarly, Yu et al. [24] reported 100% classification accuracy in adulterated buckwheat after a 76% reduction in variables, which also facilitated the identification of relevant spectral peaks (1208 and 1460 nm). Beyond accuracy, CARS has also contributed to the development of more compact and computationally efficient models. In the study by Chen et al. [66] on turmeric, the PLS model with CARS achieved an R^2^p of 0.9462 and an RPD of 4.04, outperforming the PCA and the full-spectrum model. Chai et al. [83] also emphasized that CARS reduced the number of variables in adulterated buckwheat flour to only 51 wavelengths while achieving an R^2^p of 0.9987. However, limitations have also been identified: in Sichuan samples, the CARS-based model did not outperform the full-spectrum model, highlighting its dependency on sample origin and the preprocessing method applied.

SPA has been employed as a variable selection technique in NIR spectra due to its ability to reduce collinearity among wavelengths and improve model interpretability. In studies such as that by Chen et al. [100], SPA reduced the number of variables from 198 to only 5–29, while maintaining R^2^p ≥ 0.75 in several models for adulterated rice, even achieving an RPD of 2.02, comparable to the full-spectrum model. This drastic reduction suggests advantages in computation time and model portability. However, the results also revealed that effectiveness depends on the specific mixture analyzed; in some cases—such as the KS-SNV-SPA-PLSR model—R^2^p dropped to 0.681, indicating a potential loss of useful information. Moreover, the effectiveness of SPA was inconsistent across all contexts. Moghaddam et al. [80] reported that models built with SPA-selected variables showed lower sensitivity and accuracy than full-spectrum models in adulterated protein supplements. For instance, the “adulterated” class achieved only 22% accuracy when only two variables were used, compromising the approach’s viability. Although SPA effectively reduces dimensionality, it may eliminate relevant variables when applied without proper validation. Despite these limitations, studies such as Li et al. [85] demonstrated the potential of SPA for portable devices, reducing the number of variables from 198 to only 7 in the green tea analysis. Its implementation is especially valuable when simplicity, portability, and robustness against spectral redundancy are prioritized, although its effectiveness relies on the balance between compression and information retention.

VIP is an embedded technique within PLS and PLS-DA models that allows the evaluation of each wavelength’s relative contribution to model performance, making it particularly useful for interpreting complex phenomena’ spectral basis. In the study by Casarin et al. [22], although VIP was not used to reduce variables, it played a key role in identifying relevant spectral bands associated with fraud in teff flour, enhancing chemical interpretation without directly affecting model performance. Regions with high VIP values (>1) were associated with functional groups such as CH, OH/NH, and NH, providing strong spectral evidence for differentiating authentic and adulterated samples. In contrast, Kar et al. [65] applied VIP as an active selection technique, narrowing the spectral range from 900–1700 nm to an optimized interval of 1380–1650 nm, which drastically improved the PLSR model’s performance for detecting Sudan I in adulterated turmeric. Outstanding metrics were obtained, including R^2^p = 0.979, RPD = 9.6, and RER = 24.5, indicating not only high precision but also substantial improvements in efficiency and interpretability. In contexts where spectral signals are subtle or overlapping, VIP has proven particularly effective by focusing on relevant bands and avoiding redundancy. Similarly, De Carvalho et al. [46] applied VIP in authenticity studies of Peruvian maca without explicit variable reduction, yet achieved high predictive performance (sensitivity and specificity = 1.00). The regions highlighted by VIP corresponded to absorption bands typical of adulterant compounds, such as RBPs. Although the computation time was not improved, the analysis enabled a detailed interpretation of the underlying chemical patterns.

Several advanced feature selection techniques have been explored in food adulteration analysis using NIR spectroscopy, showing notable results in terms of accuracy, variable reduction, and interpretability enhancement. For example, IWO and RF are highly effective in complex spectral contexts with high collinearity. IWO significantly improved the accuracy of an SVM model for adulterated coffee (R^2^p = 92.25%) by selecting bands associated with key compounds such as caffeine and chlorogenic acids [23]. RFrog, on the other hand, achieved the best predictive values in ginger (R^2^p = 0.9559; RPD = 4.89) using only 85 variables, standing out for its robustness against noise and redundancy [66].

Techniques such as EMCVS and RCGA have also shown strong practical applicability. EMCVS drastically reduced spectral variables (from 228 to 14–19) in cocoa shells, improving R^2^p for both portable and benchtop instruments [44]. It also enabled the identification of key functional bands, enhancing the model’s chemical traceability. RCGA, applied to turmeric, optimized models using only 10–30 wavelengths, increasing accuracy and reducing RMSEP, thus facilitating adulterant detection in portable and highly collinear environments [81].

Other methodologies, such as IRIV, SDPC-WSP, and ROBPCA, have also shown promise. IRIV maintained performance with lower computational load in green tea [85], whereas SDPC-WSP achieved accuracies above 97% in adulterated milk using only 7–22 variables [38]. When combined with one-class models, ROBPCA improved the specificity of adulterated almond flour samples, even at low concentrations [70]. Finally, techniques such as BChOA and OCPLS yielded mixed results: BChOA reduced the number of variables but performed less accurately than IWO, whereas OCPLS achieved >98% accuracy by optimizing latent components for one-class classification. Collectively, these techniques offer valuable alternatives to address the challenges of NIR analysis in food applications, each with specific advantages depending on spectral complexity and analytical objectives [23,70].

The scientific literature clearly shows that variable selection not only improves computational efficiency but also strengthens model interpretability by directly linking selected spectral regions to specific chemical compounds. The choice of technique should be aligned with matrix complexity, adulterant type, spectral quality, and the intended modeling algorithm. The combination of exploratory techniques, such as PCA, with selective algorithms, such as VIP or CARS, has proven particularly effective in NIR spectroscopy-based food authentication studies.

### 3.3. Modeling

In building chemometric models, it is essential to link each spectrum with the sample’s known authenticity or adulteration status. Once defined, the dataset is divided into subsets for calibration (training) and validation or prediction. This step is critical, as the partition quality directly influences the model’s ability to generalize beyond the calibration set and avoid overfitting [32].

Several strategies are used for dataset partitioning. Random sampling (RS) is simple but often fails to capture the data’s full variability. The Kennard–Stone (KS) algorithm [13,23], which maximizes the Euclidean distance between selected samples, and the SPXY method, which considers both spectral information (X) and response variables (Y) to achieve balanced partitions, are robust alternatives [22,99]. Recent extensions, such as kernel-based SPXY (KSPXY), further improve representativeness by accounting for nonlinear relationships [103,104]. The right partitioning approach strengthens model robustness and reliability, ensuring that NIR–chemometric tools are effective in detecting food fraud under real industrial conditions [32].

#### 3.3.1. Qualitative Classification Models

Qualitative classification in the context of food authenticity aims to discriminate between authentic and adulterated samples or between different product classes based on their spectral signatures [13,36]. In the reviewed studies, both classical statistical methods and advanced ML algorithms, including deep neural networks, have been employed—each adapted to the complexity of the evaluated matrices and adulterants. Table 3 presents the most representative techniques applied for this purpose, along with their respective advantages, limitations, and application contexts.

PLS-DA is a widely applied qualitative model for detecting food adulteration. It is noted for its balance between accuracy, robustness, and interpretative simplicity. In multiple studies, binary PLS-DA models achieved outstanding performance, with sensitivities and specificities above 94%, as reported in the authentication of adulterated paprika [82], Peruvian maca [46], and baobab powder [72]. These models were able to discriminate subtle adulterations even with a limited number of latent variables, demonstrating high efficacy as screening tools for quality control. However, the performance of PLS-DA can vary depending on the classification problem complexity. In multiclass studies or with intermediate adulteration levels (40–60%), such as those involving grated coconut [106] or buckwheat [99], a decline in accuracy and sensitivity was observed. In such scenarios, techniques such as SVM or hierarchical models—e.g., Hierarchical PLS-DA in cinnamon—proved to be more effective, particularly when combined with advanced preprocessing and feature selection strategies [13]. Nevertheless, PLS-DA remained competitive in studies such as that by Teye y Amuah [105], where it outperformed models such as RF, LDA, and SVM in the classification of adulterated rice varieties.

SVM-based qualitative classification models have demonstrated outstanding performance in food adulteration detection studies using NIR spectroscopy. Their ability to integrate with variable selection algorithms and optimized preprocessing strategies has enabled the construction of highly accurate and robust models. Yu et al. [24] reported that the Autoscale–CARS–CV–SVM model achieved 100% precision, recall, and F1 score in detecting adulteration in buckwheat flour, outperforming PLS-DA across all key performance indicators. Similarly, Chen et al. [66] achieved 100% accuracy by combining SVM with CARS in adulterated ginger and 97.91% accuracy when combined with SPA, demonstrating the versatility of SVM across different dimensionality reduction strategies.

SVM also proved to be effective in multiclass studies and in settings with significant spectral variability. Boadu et al. [69] found that an SVM model using SNV + SD preprocessing was the most effective for classifying roasted Robusta coffee, with an F1 score of 0.97, outperforming neural networks (NN), RF, and LDA. Similarly, Amsaraj et al. [81] confirmed that SVM was the most accurate model for turmeric authentication, reaching 100% accuracy with full-spectrum data and 93% with only 20 variables. Its combination with RCGA further enhanced performance even when using low-resolution portable instruments, where other models, such as XGBoost, failed to accurately classify samples.

Although in some studies, such as that by Teye and Amuah [105], SD-PLSDA outperformed SD-PLSDA in powdered adulterated rice samples, SVM still maintained high metrics (≈96–97%) and was consistently more stable than less sophisticated models such as LDA. Overall, its implementation has been particularly valuable in studies requiring high sensitivity, especially for subtle adulteration levels (<5%) or in multiclass scenarios with imbalanced classes, as evidenced by Millatina et al. [42] and Essuman et al. [98]. SVM is a highly effective, adaptable, and powerful technique for qualitative classification in complex spectral systems.

With moderate success, LDA models have been used for the qualitative classification of adulterated samples using NIR spectroscopy, standing out for their simplicity, computational efficiency, and visual interpretability. In several studies, LDA demonstrated highly positive results when combined with specific preprocessing methods. For instance, Zaukuu et al. [64] achieved accuracies above 97% in classifying adulteration in melon seed powder and up to 100% in validation sets using SG preprocessing. Similarly, Chen et al. [66] reported that LDA combined with SPA reached 100% accuracy in powdered ginger using only 13 variables, outperforming even CARS- and SVM-based configurations.

However, the performance of LDA is highly dependent on spectral preprocessing and experimental design. Boadu et al. [69] found that LDA was the least effective model compared to PLS-DA, SVM, RF, and neural networks, yielding F1 scores ≤ 0.71. Similares cases were reported in adulterated rice [105] and shrimp powder [92], where performance declined considerably in multiclass scenarios or under intermediate adulteration levels. This sensitivity was also observed by Tao et al. [41], who reported that LDA models lost accuracy relative to PLS-DA as the number of classes increased in complexity.

RF models are robust and versatile tools for the qualitative classification of adulterated samples using NIR and VIS-NIR spectroscopy. Their ability to handle high collinearity and spectral noise datasets has been validated in multiple studies. In the geographical classification of African Robusta coffee, RF achieved competitive metrics (F1 = 0.94–0.96), although it was outperformed by SVM and PLS-DA in some specific scenarios [69]. Similarly, in turmeric adulterated with starch, RF combined with SNV and PCA yielded high accuracies and greater robustness compared to other classifiers such as KNC, highlighting the importance of preprocessing in optimizing performance [71]. In studies involving complex mixtures, such as chili powder, rice, and cocoa, RF consistently maintained high performance, particularly in well-tuned configurations. Essuman et al. [98] reported high accuracy (up to 87.5%) and specificity (100%) in detecting adulteration with kola nut, although performance decreased against more challenging adulterants such as pear seed. On the other hand, Millatina et al. [42] highlighted RF’s equivalence to SVM in classifying adulteration in cocoa, with perfect accuracy and Kappa values, in addition to the interpretability advantage of tree structures for identifying key variables.

In multiclass or multi-instrument applications—such as curcumin authenticity studies—RF achieved 95–98% accuracy although it was outperformed by SVM when using lower-resolution spectral devices [81]. Although RF does not always lead in absolute accuracy, its balance of performance, stability, and interpretability makes it a reliable classifier in environments with high spectral variability, especially when chemical traceability and informative band selection are required.

SIMCA models and their variants, such as DD-SIMCA and PCA-SIMCA, have shown remarkable utility in food authentication and detection of adulteration using NIR spectroscopy. In the study by Netto et al. [78], DD-SIMCA combined with FD preprocessing achieved 100% sensitivity and specificity for almond flour adulteration, outperforming the classical SIMCA and OCPLS. In portable applications, OCPLS + MicroNIR exhibited strong performance although SIMCA with SNV showed low specificity (72.2%). In binary classification contexts, DD-SIMCA proved to be the most effective model for detecting cinnamon adulteration with nutshells, surpassing PLS-DA in both sensitivity and specificity when dealing with challenging adulterated samples [13]. Similarly, FT-IR + DD-SIMCA achieved better performance than portable NIR in studies involving cumin, underscoring the critical role of instrumentation and preprocessing in model performance [93].

The hierarchical SIMCA approach is effective in more complex scenarios. Ejeahalaka and On [78] reported high accuracy with three-level models applied to milk powder adulterated with melamine, successfully detecting fraud at concentrations as low as 0.01%. Similarly, SIMCA models applied to fortified milk accurately distinguished between fresh and aged samples [108], and in black pepper and cumin, SIMCA achieved higher sensitivity than supervised methods like PLS-DA, though with moderate specificity [109]. Finally, Rukundo y Danao [74] demonstrated that the effectiveness of PCA-SIMCA heavily depends on the calibration design; including adulteration levels within the model is essential to ensure robustness against real adulterated samples.

The KNN model has variable performance in food authentication tasks using NIR spectroscopy. In the study by Moghaddam et al. [80], KNN achieved results comparable to PLS-DA in binary classification of protein supplements, reaching 100% sensitivity and specificity when appropriate preprocessing methods, such as SNV or MSC, were applied. However, its performance was considerably less stable in multiclass classification, with significant drops in sensitivity (down to 60%) when SNV-DT combinations were used without parameter optimization. This highlighted the higher sensitivity of the model to preprocessing choices compared to more robust classifiers. In contrast, in the work of Lanjewar et al. [71], KNN was the least effective algorithm for detecting starch-adulterated turmeric, yielding precision metrics below 50% under schemes like SG-PCA. Compared with models such as Random Forest or Extra Trees, which performed remarkably well with SNV-PCA, KNN revealed substantial limitations in contexts with high spectral collinearity and multiple adulteration levels. These findings suggest that while KNN can be competitive in simple, well-preprocessed tasks, it is not a reliable option for complex or multiclass scenarios.

CNNs in food adulteration detection have shown promising results. Ku et al. [97] reported that the CNN model achieved an overall classification accuracy of 92.8% for adulterated cinnamon samples, exhibiting outstanding performance for pure classes and low-level adulteration (10%). However, performance declined for intermediate adulteration levels (20% and 30%), indicating reduced sensitivity to mixtures with subtle spectral differences and suggesting the need for optimization to improve discrimination in borderline concentrations. In a more recent study, Sadeghi et al. [39] integrated the NIR spectra with the RGB image data in a CNN model to detect adulterants in turmeric, achieving high classification metrics. Sensitivity ranged from 0.774 (at 20% adulteration) to 1.000 (for 0% and 25% classes), whereas specificity remained high across all levels (0.967–1.000). The model performed particularly well at the extremes of the adulteration range (0% and 30%), where the spectral differences were more pronounced. The confusion matrix and receiver operating characteristic (ROC) analysis confirmed that most samples were correctly classified with minimal errors, validating the CNN model’s ability to handle complex spectral scenarios with high precision.

Emerging classification and variable selection techniques include DTC, ETC, XGBoost, OCPLS, OPLS-DA, SDPC, and WSP. Their performance varies depending on the type of adulterant, preprocessing strategy, and spectral characteristics of the dataset.

For instance, DTC and ETC achieved high accuracy when applied to preprocessed Vis-NIR spectra and reduced with PCA for detecting starch-adulterated turmeric. According to Lanjewar et al. [71], the SNV + PCA + RFC or ETC combination outperformed other approaches, while KNN yielded a notably lower performance. These results underscore the value of tree-based classifiers in multi-concentration settings with well-structured spectral data.

XGBoost demonstrated excellent performance in detecting commercial curcumin adulteration, reaching 95–98% accuracy [81]. However, its efficacy significantly declined with low-quality instrumental spectra, indicating greater sensitivity to the signal-to-noise ratio compared to SVM or RF although its regression capability was highlighted.

The OCPLS model, designed for one-class classification, achieved 100% sensitivity and 98.3% specificity using MicroNIR (Viavi Solutions, Scottsdale, AZ, USA) and first derivative preprocessing, emerging as the most effective technique among portable devices for almond flour authentication [70]. Conversely, OPLS-DA, applied to black pepper and cumin adulteration detection, offered high specificity but poor identification of genuine samples, suggesting its complementary use alongside SIMCA [109].

Additionally, when combined with kNN, SDPC and WSP drastically reduced the number of wavelengths (from 1050 to just 1–22) without compromising accuracy. Yuan et al. [38] reported RARP and RARV values exceeding 96%, even in regions with spectral overlap, highlighting the potential of these techniques to construct compact and efficient models with strong spectral discrimination capabilities.

The choice of qualitative classification model is influenced by multiple factors, including matrix complexity, adulterant type, spectral quality, dataset size, and specific analysis goals (e.g., screening, validation, and authentication). While classical statistical models such as PLS-DA and LDA remain valuable under controlled conditions, advances in ML and DL offer more flexible and scalable solutions for real-world environments—especially when paired with robust preprocessing and variable selection strategies. The growing use of hybrid approaches and ensemble classifiers reflects a trend toward smarter and more adaptive systems for non-destructive control of food authenticity.

#### 3.3.2. Quantitative Prediction Models

Regression models applied to NIR spectra aim to accurately and robustly estimate the adulterant concentration by capturing both linear and nonlinear relationships within highly collinear datasets. The reviewed literature reveals two predominant approaches: ML algorithms and DL methods, each with specific advantages depending on the application context. Table 4 summarizes the most commonly used models for quantitative prediction in food powder authenticity studies, outlining their typical applications, strengths, and limitations.

PLS and its variants are highly effective tools for the detection and quantification of adulterants in food products, especially when combined with NIR spectroscopy. Across a wide range of matrices such as rice, flour, supplements, dairy products, and spices, PLS models have achieved R^2^ values above 0.97 and low prediction errors (RMSEP < 3%), enabling accurate estimation even at adulteration levels equal to or greater than 1% [14,22,42]. Particularly remarkable results were obtained in matrices with good spectral homogeneity and optimally preprocessed spectra, such as grape seed extract [73] and adulterated whey powder [80].

Extensions of PLS, such as iPLS and siPLS-PLSR, have shown additional advantages by enabling the selective use of relevant spectral intervals, reducing collinearity, and improving model interpretability. For instance, in mixtures of fats adulterated with urea, iPLS outperformed the conventional model in terms of accuracy and parsimony [78]. However, the most robust model was siPLS, which synergistically combined multiple intervals to enhance predictive capability, as demonstrated in studies on adulterated maca and premium rice [91,105]. In these cases, siPLS consistently outperformed both PLS and iPLS across all performance indicators.

However, the effectiveness of PLS models also strongly depended on the spectral quality, adulterant type, and preprocessing technique employed. For example, in complex mixtures such as whey protein adulterated with various nitrogen-based compounds, PLS models combined with SNV or MSC achieved R^2^ > 0.99 and RPD values > 10 [68,95]. In contrast, in matrices with greater nonlinear complexity—such as rice with varying particle sizes [100] or wheat adulterated with surrogate compounds [83], techniques such as SVR or neural networks outperformed PLS models, although they remained competitive under controlled conditions.

PLS and its variants represent a reliable, scalable, and highly accurate strategy for quantifying food product adulterants. Their versatility has been demonstrated across various analytical and instrumental settings, ranging from portable spectrometers to laboratory-grade equipment. However, optimal performance requires careful selection of preprocessing methods, a combination of relevant spectral variables, and robust cross-validation. In contexts where nonlinear relationships or high spectral heterogeneity predominate, combining PLS with complementary techniques, such as SVR or hybrid models, may be beneficial to achieve greater accuracy [39,77].

SVR has demonstrated competitive performance in detecting and quantifying food adulteration using NIR spectroscopy, particularly in contexts with high spectral complexity. In the case of high-value adulterated rice, the SPXY-MSC-CV-SVR model achieved an R^2^p of 0.9467 and an RPD of 4.3287, outperforming PLSR even when combined with variable selection techniques, such as SPA or CARS [100]. Similarly, the combination of SVR and IRIV outperformed PLSR in terms of accuracy, efficiency, and predictive stability in the analysis of green tea using portable spectroscopy, achieving an RPD of up to 17.32 for sugar adulteration in tea [85].

However, the SVR performance varies depending on the matrix type and instrumental configuration. SVR was more effective than PLSR for predicting GTE in grape seed extract using the NIR-S-G1 device (InnoSpectra Co., Hsinchu, Taiwan), but showed inferior performance when predicting specific compounds, such as catechin and epicatechin, with portable instruments [73]. In studies involving turmeric adulterated with Sudan I, SVR models were consistently outperformed by PLSR across all metrics [65], indicating limitations in scenarios where the spectrum-concentration relationship is essentially linear or where the spectra exhibit high redundancy.

Additionally, in a study on buckwheat flour adulteration, the effectiveness of chemometric models was found to depend on the samples’ geographical origin. While SVR models were more accurate in samples from Sichuan and Shanxi—regions characterized by greater spectral complexity—achieving R^2^p values above 0.9957, the PLSR model showed the best performance in samples from Inner Mongolia [83]. In contrast, a multispectroscopic analysis of turmeric showed that SVR yielded intermediate performance levels, outperforming ensemble models such as Random Forest and XGBoost [81].

SVR is a powerful and adaptable technique that is especially useful in nonlinear contexts or with complex spectra. However, its effectiveness heavily depends on preprocessing, variable selection, and data quality, making it advisable to compare it with other ML techniques and classical linear models for each specific application.

Compared with other multivariate methods, PCR has generally shown inferior results in studies on the detection and quantification of food adulteration using NIR and Vis-NIR spectroscopy. In the case of turmeric adulterated with Sudan I, PCR exhibited a higher root mean square error (RMSE) than PLS, and although the differences were not statistically significant according to the Diebold-Mariano test, they were significant according to the F-test, casting doubt on its comparative effectiveness [65].

In applications aimed at predicting adulteration levels in flours, such as mixtures of brown rice with white rice, PCR proved to be less effective than PLSR, showing lower accuracy and robustness against spectral variability [77]. In studies on legumes—for example, detecting pea and bitter vetch flour in mixtures with chickpea flour—PCR performed the worst, with errors two to three times higher than those obtained by MPLSR, indicating poor fitting and generalization capacity [86].

Similarly, PCR was the least effective model in the prediction of adulteration with corn flour in chickpea flour across all evaluated metrics. Its lower RPD and higher prediction error were clearly outperformed by more sophisticated methods, such as MPLSR and PLSR, confirming its limited performance in scenarios requiring high sensitivity and precision [87]. Despite its simplicity, these findings suggest that PCR is not the best choice for food authenticity control applications—especially when more robust and accurate alternatives are available.

The performance of the RFR algorithm in food authenticity studies has been variable, with results ranging from outstanding to significantly limited depending on sample type, spectral preprocessing, and variable selection strategy. In a study by Lanjewar et al. [71], RFR demonstrated exceptional performance in detecting starch-adulterated turmeric, achieving remarkable metrics such as a validation R^2^ of 0.999, an RMSEv of 0.391 mg/w, and an RPD of 92.3 after combining SNV preprocessing and dimensionality reduction via PCA. These values significantly outperformed those of other models, such as DTR and KNR, establishing RFR as a reliable tool for detecting adulteration.

In a multi-instrument comparative analysis of commercial turmeric authenticity, Amsaraj et al. [81] reported that RF achieved prediction values (RP) ≥ 0.98 on benchtop instruments using reduced variable sets, demonstrating its ability to maintain accuracy with optimized models. However, in other cases, such as the study by Behera et al. [107] on turmeric adulterated with exhausted turmeric, RF showed lower performance compared to simpler techniques, such as linear regression, exhibiting higher standard deviation and lower explanatory power. This reflects that, while RFR is a powerful tool in scenarios with good preprocessing and optimal configuration, its effectiveness may decline when inadequate variable selection combinations are applied or when uncontrolled spectral noise conditions are encountered.

In the analysis of regression models applied to the detection and quantification of adulterants in food matrices using NIR spectroscopy, clear performance differences are evident depending on the architecture employed and the type of preprocessing applied. For example, when applied to Micro-NIR spectra, convolutional neural network models such as GoogleNet, ResNet, S-AlexNet, and Simple CNN performed remarkably well in detecting adulteration in coconut milk, achieving R^2^ values of 0.999 and RPD values above 30, positioning them as highly effective tools for complex and nonlinear scenarios [84]. However, their performance was compromised when using lower-resolution spectra, such as FT-NIR, highlighting the importance of the type of spectral data used.

Conversely, classical models, such as BPNN, consistently demonstrated high performance in multiclass scenarios, outperforming SVR and PLSR with R^2^p values above 0.97 and RPD > 4 in studies on rice adulteration [100]. Similarly, hybrid architectures, such as CNN-Regression, were also competitive, although they showed lower predictive robustness compared to PLSR when using NIR spectra without targeted variable selection [39]. In contrast, tree-based algorithms such as Random Forest Regressor (RFR) and Extra Trees Regressor (ETR) achieved outstanding performance in detecting starch-adulterated turmeric, with validation R^2^ up to 0.999 and RPD of 92.3 [71], although their performance was inferior in less structured scenarios, such as those evaluated by Behera et al. [107], where linear regression outperformed them.

Ensemble-based models (e.g., XGBoost, Gradient Boosted Trees, and Ensemble Tree Regression) have also demonstrated strengths in turmeric and cocoa authentication tasks, especially when combined with proper variable selection and spectral preprocessing. XGBoost, for instance, achieved RP = 0.9999 and a RMSEP of only 0.0096 using FT-NIR platforms, ranking as the best-performing model among more than five algorithms [81]. In contrast, simpler algorithms, such as Elastic Net, LASSO, and Ridge, yielded intermediate results. Although LASSO and ENet without Boruta reached RPD > 13 [42], they did not outperform more advanced nonlinear models, such as BPNN or XGBoost.

Finally, ELM, GA-PLS, MLP, and MLR architectures produced acceptable results but were generally inferior in terms of precision and comparative stability. Their utility appears to be limited to low-complexity spectral scenarios or as benchmark baseline models. In some cases, simple linear regression was surprisingly competitive when the spectrum–concentration relationships were well-defined and linear [107].

The regression model should consider spectral complexity, adulterant type, matrix characteristics, and sample size. Statistical methods, such as PLSR, offer a solid and interpretable foundation, whereas machine learning and deep learning algorithms provide greater flexibility and accuracy in nonlinear or multiclass scenarios. The emerging trend toward hybrid and optimized food quality control models suggests a technical evolution pathway that combines analytical robustness with operational scalability.

### 3.4. Validation

Model validation is a critical step in chemometric workflows, ensuring that predictive models are both reliable and generalizable [111]. This process can be addressed through two complementary approaches: internal and external validation.

#### 3.4.1. Internal Validation

Internal validation ensures that predictive models are not limited to the calibration set but can be generalized to unseen data. Cross-validation (CV) is the most common approach in NIR-based fraud detection, which divides the dataset into subsets for iterative training and testing, reducing overfitting. Variants include leave-one-out (LOOCV), which is robust for small datasets, as applied by Zaukuu et al. [64] with a leave-one-sample-out strategy and by Luckas et al. [68] using leave-one-replicate-out. Another widely used method is K-fold CV, which balances efficiency and robustness by cycling through K partitions. For instance, Boadu et al. [69] emphasized its broad use in chemometrics, while Castro et al. [76] applied a 5-fold scheme to validate adulteration models.

Simpler alternatives, such as hold-out validation, exist, where the dataset is split once from the same batch into calibration and prediction subsets. Tao et al. [41] demonstrated this with a two-thirds/one-third division. Although less exhaustive, it still provides valuable insights under controlled conditions. Collectively, these strategies highlight the flexibility of internal validation methods and their role in ensuring the reliability of NIR–chemometric models for powdered food authentication.

#### 3.4.2. External Validation

External validation is considered the most rigorous approach for assessing the predictive ability of chemometric models because it evaluates performance on independent data not used during calibration. This strategy simulates real industrial conditions, in which models must reliably predict new samples from different replicates, batches, or explicitly separated datasets. For instance, Lukacs et al. [73] applied a rotational partitioning scheme in seed powders, allocating two-thirds of replicates for training and one-third for testing. The process was repeated to ensure that all samples were included in both calibration and validation. In protein powders, the same authors implemented an independent test set, reserving one replicate for validation while calibrating with the remaining ones, thus guaranteeing a strict separation between training and prediction data.

A classical approach to external validation involves dividing the entire dataset into two independent subsets. Oliveira et al. [82] exemplified this by splitting 315 samples; 171 into a calibration set and 144 into a prediction set [, allowing the evaluation of both the PLS-DA and PLSR models with completely unseen data. These examples illustrate how external validation strengthens the credibility of NIR–chemometric studies by confirming model robustness and generalizability beyond the internal checks provided by cross-validation.

### 3.5. Metrics Evaluation

Different evaluation metrics are applied to assess the performance and reliability of chemometric models depending on whether the task is qualitative (classification) or quantitative (regression). For classification models, metrics such as accuracy, sensitivity, specificity, precision, and F1-score provide insight into the effectiveness of the model in discriminating between authentic and adulterated samples (Table 5). In contrast, regression models are evaluated using statistical measures like R^2^, RMSE, bias, SEP, RPD, and RER, which reflect prediction accuracy, error distribution, and practical utility (Table 6).

### 3.6. Software Packages for NIR Chemometric Analysis

NIR spectra generate large volumes of data, and their chemometric analysis requires software capable of efficiently handling this information. Such tools must allow the implementation of various preprocessing techniques, feature selection methods, robust classification and regression models, as well as the generation of graphical reports, tables, and file management functions that support the proper interpretation of the spectra. A wide range of software packages, both commercial and open-source, are available to meet these needs, offering different levels of complexity, flexibility, and cost. The choice of software largely depends on the user’s background or programming experience, as well as the available economic resources. Table 7 summarizes the most widely used packages, highlighting their main features, advantages, and limitations.

Regarding the chemometric analysis, there is no universal recipe for the optimal combination of preprocessing techniques, feature selection methods, and chemometric models to detect adulterants in food products using NIR spectra. The performance of each approach depends on several factors, including the matrix under evaluation, the type of device used, the identification of spectral interferences, and the proper implementation of the chemometric models. Preprocessing techniques help correct interferences related to the sample, instrument, or packaging material, particularly in non-invasive measurements. Meanwhile, feature selection simplifies the models, reduces computational load, and enhances both efficiency and accuracy. Qualitative classification and quantitative prediction models deliver varying levels of performance depending on the analytical goal. Section 6 (Table 10) summarizes the main performance metrics reported across the reviewed case studies to support practical comparison, enabling the identification of methodological strengths and areas for improvement in future research.

## 4. Common Adulterants and Their Impact on Nutritional Quality and Health

The addition of adulterants to powdered foods is often driven by economic motivations, local availability, and visual similarity, which makes them difficult to detect. Frequently, matrices with similar appearance, density, or particle size to the authentic ingredients are used, posing significant risks not only to food authenticity but also to the final product’s safety and nutritional quality. Table 8 summarizes the main adulterants identified in powdered foods based on recent NIR spectroscopy and chemometric studies.

Powdered milk and protein supplements, particularly those intended for infants and athletes, have been adulterated with nitrogen-based compounds, such as melamine and urea, to simulate a higher protein content. This practice is especially dangerous because melamine can induce the formation of insoluble crystals in the urinary tract, causing severe nephropathies and even death, as evidenced by the 2008 scandal in China, which affected more than 300,000 babies. Urea addition also overloads hepatic and renal metabolism, posing a critical risk to infants and immunocompromised individuals [67,68,108].

Likewise, the substitution of sweet almonds with bitter almonds introduces dangerous levels of amygdalin, a glycoside that releases cyanide, a lethal toxin, upon metabolism in the body. Although appearance and taste may not differ significantly, the toxicity of this adulterant can trigger acute symptoms such as nausea, vomiting, respiratory distress, and, in severe cases, death by systemic poisoning. This is particularly concerning in products targeted at children or the elderly [63].

Unauthorized synthetic dyes, such as Sudan I and Metanil Yellow, detected in adulterated turmeric samples possess carcinogenic and hepatotoxic properties. The International Agency for Research on Cancer has classified Sudan I as a possible human carcinogen, while Metanil Yellow has been associated with liver damage and hematological disorders. Chronic exposure to these compounds may induce cellular mutations, hepatic metabolic disruption, and DNA damage, posing a serious risk especially for populations with high turmeric consumption due to cultural or therapeutic reasons [65,74].

The adulteration of black pepper with papaya seeds may appear harmless from a visual perspective; however, these seeds contain compounds such as benzyl isothiocyanate, whose concentrations have not been fully characterized in this context. Prolonged exposure could induce toxic effects, which have not yet been fully documented, but can potentially cause gastrointestinal discomfort or side effects when consumed in large amounts. Despite its lower lethality, adulteration constitutes deception and a potentially unquantified health risk [127].

The addition of walnut, pecan, and peanut shells to cumin poses a relevant sanitary risk, especially for individuals allergic to tree nuts. Although these residues are not toxic per se, they may contain potent allergens and contaminant compounds if not properly processed. Moreover, this adulteration degrades the functional value of cumin, altering its phytochemical profile and reducing its essential oil content, affecting its culinary and therapeutic efficacy [93].

Adulteration of nutraceutical products, such as powdered maca, by substituting it with cheap starches, such as rice or rice bran, presents a subtle yet significant threat. Although these ingredients are not toxic, they dilute the bioactive compounds characteristic of maca—such as aramides, macaense, and glucosinolates—which are responsible for its energizing, adaptogenic, and hormone-regulating effects. This substitution not only reduces the therapeutic value of maca but also alters its nutritional and sensory profile, thereby compromising its authenticity and expected efficacy in functional or medicinal contexts [46].

Similarly, adulteration in matrices such as coffee with soy [23] or pseudocereals such as buckwheat [99] and teff with common wheat [22] compromises both sensory attributes and safety for individuals with allergies or celiac disease. Although these substitutions are not generally lethal, they pose significant risks to sensitive groups, potentially triggering responses ranging from mild allergic reactions to anaphylaxis.

Adulterants in powdered foods, such as melamine, Sudan I, or bitter almonds, represent serious health risks that often go unnoticed because of their visual similarity to the authentic product. In this context, NIR spectroscopy provides a valuable solution by enabling the rapid, nondestructive quantification and detection of these compounds without the need for reagents. However, its application must go beyond basic quality control. It is crucial that it focuses on identifying adulterations with high health impact and integrates it into surveillance systems that prioritize consumer protection. In doing so, NIR can evolve into a strategic tool for detecting food fraud and preventing real public health threats.

## 5. NIR Devices: Capabilities and Limitations for Food Fraud Detection

In recent years, NIR spectroscopy devices have undergone significant evolution, transitioning from highly specialized benchtop instruments to portable solutions designed for on-site analysis without compromising analytical accuracy [13,63]. The growing demand for real-time quality control across the food supply chain has driven this transformation, particularly for products vulnerable to adulteration, such as spices, flours, and plant-based powders [70,95].

Compact and low-cost instruments have democratized access to this technology, facilitating its integration into industrial and field settings. However, challenges remain—particularly in terms of spectral resolution, environmental interferences, and low-concentration adulterant detection limits. Therefore, understanding the technical specifications, capabilities, and limitations of the different NIR devices currently available is essential to select the most appropriate tool for a given analytical context. Table 9 summarizes the key characteristics of NIR instruments reported in the recent literature.

### 5.1. Portable NIR Detection Devices

#### 5.1.1. Technical Specifications

Near-infrared (NIR) spectroscopy-based portable devices have gained prominence as versatile analytical tools for the rapid and nondestructive analysis of powdered food products. These instruments are optimized to operate within a limited spectral range, commonly between 900 and 1700 nm, which includes overtone and combination bands associated with key functional groups, such as –OH, –CH, and –NH, responsible for the characteristic interaction with NIR radiation in organic compounds present in food matrices [13,95].

Despite their compact size, these devices exhibit significant variability in terms of spectral dimensionality. Some instruments designed for low-cost or mass-use applications operate with simplified spectra of 10–20 distinct bands, strategically selected to maximize class discrimination [65]. In contrast, the more advanced versions offer up to 100 wavelengths, providing greater spectral information richness. However, their spectral resolution, typically between 10 and 20 nm, is lower than that of benchtop systems, which may limit their ability to detect subtle changes in complex mixtures [63,70].

Regarding optical configuration, portable devices predominantly employ diffuse reflectance geometries, ideal for powdered samples due to their ability to capture signals from irregular surfaces without prior preparation [95]. Miniaturized halogen lamps or broadband NIR LEDs are the most common light sources, while detectors, mostly based on InGaAs technology, offer efficient response within the operational range with reduced energy consumption [13]. Compatibility with various sample formats —from powders directly applied to the optical window to samples in translucent plastic containers— adds operational flexibility, although it introduces challenges related to sample homogeneity and positioning consistency [65].

#### 5.1.2. Operational Advantages

The main strength of portable devices is their deployability at any point in the supply chain, from warehouses to farms. This feature, combined with their high analysis speed —usually under one minute per sample—makes them ideal tools for rapid and preliminary decision-making [13,63].

Moreover, their minimal sample preparation requirements —no fine grinding, liquid homogenization, or prior dissolution needed—are crucial in contexts where time and resources are limited [23,63]. This technical accessibility has facilitated their incorporation into production, commercial, and logistics environments, requiring minimal user training.

A recent study demonstrated that the precision of a portable NIR device can be competitive with that of benchtop equipment in certain matrices. For example, a Micro-NIR portable spectrometer outperformed a benchtop FT-NIR instrument in detecting adulteration in coconut milk, achieving RPD values up to 39.35 and RMSE below 0.4%, even without complex spectral preprocessing [84]. These findings reinforce the usefulness of the proposed method as a cost-effective, adaptable, and efficient screening technology for real-time non-destructive control.

#### 5.1.3. Limitations

Despite their operational advantages, portable devices have structural limitations that may compromise analytical performance. The main drawback is their low spectral resolution (typically between 10 and 20 nm), which restricts their ability to identify low-level adulterations or highly complex mixtures [43]. This limitation also results in the loss of key spectral bands, reducing precision compared to benchtop equipment.

Additionally, their performance can be affected by environmental conditions, such as intense natural light, dust, or mechanical vibrations, which generate spectral noise and reduce reproducibility. For example, Song et al. [14] reported difficulties with portable devices due to environmental interference, although with appropriate preprocessing (SG + SNV) and PLSR models, R^2^p values up to 0.991 were achieved for protein prediction in dietary supplements.

Comparative studies reinforce this gap: Lukacs et al. [73] found that PLSR models built with spectra from a benchtop NIR spectrometer (XDS) significantly outperformed those generated with portable devices in terms of accuracy (R^2^p = 0.993; RMSEP = 0.264%), which was attributed to their higher resolution and extended spectral range. Similarly, the limited capacity of portable devices to implement complex multiclass models or recalibrate against variations in sample composition reduces their applicability in exploratory studies or environments with high batch variability [63]. Therefore, while portable NIR devices are suitable as screening and rapid monitoring tools, their use should be understood as complementary—not substitutive—to high-precision spectrometers in regulatory contexts, official validation, or advanced research.

### 5.2. Benchtop NIR Devices

#### 5.2.1. Technical Specifications

Benchtop devices based on near-infrared (NIR) spectroscopy represent the reference standard in terms of analytical capacity, precision, and spectral stability. Unlike their portable counterparts, these instruments operate over an extended spectral range, typically covering 900–2500 nm, encompassing the full set of overtone and combination bands in the NIR region associated with O–H, C–H, and N–H bonds [43,97,108]. This broad spectral coverage enables the detection of secondary signals from minor compounds, which is essential in authentication studies and quality analysis.

The spectral resolution of these devices is generally superior to 2 nm, and their spectral dimensionality can reach several hundred wavelengths, providing high-density continuous spectra that support precise chemometric modeling [13]. These instruments incorporate sophisticated optical systems that ensure uniform light distribution in solid samples and minimize scattering effects [24,40]. Because of this advanced infrastructure, benchtop spectrometers are highly adaptable to multi-analyte studies, capable of simultaneously quantifying multiple chemical components and detecting complex adulteration profiles using robust and stable models over time [38,95,108].

#### 5.2.2. Technical Advantages

The main technical advantages of benchtop instruments are their high precision and reproducibility—critical aspects for generating robust predictive models [67]. These devices can produce reference spectra, i.e., spectral profiles with low noise levels and high resolution, which can be used as standards for cross-validating models developed with other types of instruments [44,68].

Recent studies have confirmed this analytical superiority. For instance, Lukacs et al. [68] demonstrated that models developed using NIRS6500 (FOSS NIRSystems, Inc., Silver Spring (Laurel), MD, USA) spectra achieved significantly higher accuracy (R^2^CV = 0.96; RMSECV = 0.15 g/100 g) than those generated with portable devices, particularly in melamine detection. In another study, PLSR models based on spectra from the 1100–2250 nm range reached R^2^P values of up to 0.993 and RMSEP as low as 0.264%, attributable to the instrument’s higher spectral resolution and coverage of critical bands [73].

Similarly, Amsaraj et al. [81] reported that in curcumin detection in turmeric, spectra obtained from benchtop devices allowed the development of models with RP = 0.9999 and RMSEP = 0.0096 using XGBoost, outperforming the performance of portable versions. Oliveira et al., [44] also demonstrated a clear advantage for benchtop devices, achieving R^2^P = 1.00 and RMSEP = 0.21 in the prediction of impurities in cocoa husks using PLSR models. Finally, Netto et al. [70] demonstrated that a DD-SIMCA model built from benchtop spectra achieved 100% sensitivity and specificity for adulteration levels ≥5%, confirming its utility in regulatory applications.

Additionally, these instruments show greater compatibility with advanced multivariate analysis, facilitating the application of exploratory techniques such as PCA or PLS-DA, as well as the training of supervised classification algorithms in multiclass and multivariable scenarios [67,81]. This analytical capacity, combined with their instrumental stability, enables the development of robust and transferable models for authenticity monitoring in highly complex food products.

#### 5.2.3. Limitations

Despite their analytical advantages, the logistical and operational limitations of benchtop spectrophotometers restrict their applicability in direct production contexts or field inspections [14]. One of the most evident barriers is their high acquisition and maintenance costs, which hinders their adoption in resource-limited supply chains or small-scale industries [70]. Furthermore, their stationary nature prevents mobile use, as they require controlled environmental conditions, stable electrical infrastructure, and trained operators for proper functioning [13]. These requirements limit their utility in scenarios such as sanitary inspections, point-of-sale control, or on-site authenticity verification.

Finally, benchtop devices offer less operational flexibility than portable instruments. Each analysis typically requires rigorous sample conditioning, precise cleaning of the reading cell, and manual adjustment of measurement parameters, which significantly increases the analysis time. In contrast, portable devices enable faster evaluations with minimal preparation, making them more suitable for immediate decision-making in the field, albeit with generally lower precision [44,68].

In addition to the technical differences between portable and benchtop NIR instruments, highlighting their performance in real-world applications is important. Table 10 presents numerous studies in which both types of devices have been used to detect adulterants in powdered foods. Portable instruments have shown good performance in practical scenarios, such as the detection of metanil yellow in turmeric [74] or nuts in cumin [93], with accuracies above 90%. Benchtop devices have demonstrated higher accuracy in more complex matrices, such as in the detection of dyes in turmeric [81] or impurities in cocoa [42], reaching R^2^p values close to 1.00 and minimal errors. These cases illustrate how the choice of instrument should be based not only on technical criteria but also on the context of use, adulterant type, and required analytical rigor.

## 6. Selected Case Studies

NIR spectroscopy has proven to be a versatile, rapid, and accurate analytical strategy for detecting adulterants in powdered foods. Through the systematic analysis of various studies—organized by sample type—it is possible to identify patterns in equipment usage, common adulterants, applied chemometric models, and achieved detection levels can be identified. These data are detailed in Table 10.

**Table 10:** Applications of NIR spectroscopy and chemometric models in adulterant detection in powdered foods classified by food category, adulterant, and type of model applied.

Category	Matrix	Adulterant	Device Type	Spectral Range	Chemometric	Results	Source
Spices and seasoning powders	Turmeric (*Curcuma longa*)	Corn, rice, and wheat	Portable	833–2500	SD-DT-SNV-PCA-CNN-1D	R^2^p = 0.848; MSEp = 16.017	[39]
	Turmeric (*Curcuma longa*)	Superior quality starch	Benchtop	400–1050	SNV-PCA-RFR	R^2^p = 0.999; RMSEp = 0.391	[71]
	Turmeric (*Curcuma longa*)	Carcinogenic dye of Sudan I (1-[(2,4-dimetilfenil)azo]-2-naftalenol)	Benchtop	900–1700	VIP-PLSR	R^2^p = 0.979; RMSEp = 0.0093	[65]
	Turmeric (*Curcuma longa*)	Other Curcuma species, cheap starches, sawdust, and chemical adulterants: metanil yellow, lead chromate, Sudan red, acid orange, aniline, and chalk powder.	Benchtop	868–2540	SNV-RCGA-XGBoost	R^2^p = 0.999; RMSEp = 0.0096	[81]
	Turmeric (*Curcuma longa*)	Metanil Yellow (illegal dye)	Portable	780–2500	SG-PCA-SIMCA	Accuracy = 97.4%	[74]
	Turmeric (*Curcuma longa*)	Spent turmeric	Portable	400 -1000	PCA-SVM	Accuracy > 90.5%	[107]
	Cinnamon	Shells of peanut, pecan, and walnut	Portable	900–1700	Hierarchical PLS-DA	Sensibilidad = 0.8–0.9	[13]
	Cinnamon	Coffee and corn bran are used	Benchtop	1100–2000	SG-PLSR	R^2^p = 0.994; RMSEp = 0.031	[75]
	Cinnamon	Hazelnut	Benchtop	1000–2500	CNN	Accuracy = 92.8%	[97]
	Jengibre (*Zingiber officinale*)	Bean	Benchtop	1000–1700	MSC-PLS	Rp = 0.99; RMSEp = 0.65	[40]
	Jengibre (*Zingiber officinale*)	Corn	Portable	900–1700	SG-SNV-Rfrog-PLSR	R^2^p = 0.956; RMSEp = 0.022	[66]
	Paprika	Pedicel, peduncle, and seed cake	Benchtop	1100–2500	SG-PLSR	R^2^cv = 0.978–0.971; RMSEcv = 5.76–6.23	[76]
	Paprika	Potato and acacia gum; annatto or achiote	Benchtop	900–1700	SNV-FD-PLSR	R^2^p = 0.968; RMSEp = 0.0017	[82]
	Chili pepper *(Capsicum annum*)	Avocado seed and kola nut	Portable	740–1070	PLS-DA	Accuracy = 91.25%	[98]
	Cumin (*Cuminum cyminum* L.)	Walnut, peanut, and pecan	Portable	900–1700	PLSR	RPD = 3.61–4.39; RMSEp = 0.003–0.006	[93]
	Black pepper (*Piper nigrum*) and cumin extract	Cassava, corn	Benchtop	1100–2500	Autoscaling-PLSR	R = 0.95; RMSE = 0.003–0.005	[109]
Cereals and Pseudo-cereal powders	Tartary buckwheat (*Fagopyrum tataricum*)	Whole wheat, oats, soy, barley, and sorghum	Benchtop	900–1700	Autoscales-CARS-SVM	Accuracy = 100%; F1 score = 100%	[24]
	Tartary buckwheat (*Fagopyrum tatari-cum*)	Common buckwheat (*Fagopyrum esculentum*)	Benchtop	900–1700	SNV-DT-CARS-PSO-SVR	R^2^p = 0.99; RMSEp = 0.0002	[83]
	Durum wheat (*Triticum durum*)	Common wheat (*Triticum aestivum*)	Benchtop	900–1650	Baseline-PLSR	R^2^p = 0.867; RMSEp = 0.009	[110]
	Commercial wheat (Five Roses, Canadá)	Cassava	Portable	1200–2100	SG-FD-PLS-DA	Accuracy = 93.83%	[41]
	Wheat	(1) Talc powder and (2) benzoyl peroxide	Benchtop	680–2600	(1) CARS–PLSR (2) SNV-PLSR	(1) R^2^p = 0.996; RPD = 15.35 (2) R^2^P = 0.964; RPD = 5.42	[88]
	Rice var. (Wuchang, Thai fragrant)	Rice var. South Japonica, Song Japonica, Jiangxi silk, and Yunhui	Benchtop	900–1700	Back Propagation Neural Network (BPNN)	R^2^p = 0.973; RMSEp = 0.071	[100]
	Brown rice	Rice	Portable	400–1000	SG-PLSR	R^2^p = 0.96; RMSEp = 0.004	[77]
	Premium Jasmine 85 variety rice	Rice var. Agra (lower demand variety)	Portable	740–1070	Si-PLS	R^2^p = 0.936; RMSEp = 0.156	[105]
	Teff (*Eragrostis tef*)	Rice, oats, whole wheat, and rye	Benchtop	1100–2500	MSC-SD-PLSR	R^2^p = 0.974; RMSEp = 0.07	[22]
	Quinoa (*Chenopodium quinoa* Willd)	Wheat, rice, corn, cassava, and buckwheat	Portable	900–1700	VIP-PLSR	R^2^p = 0.98; RMSEp = 0.0002	[102]
powdered dairy products	Whey protein concentrate (WPC), vanilla flavor	Maltodextrin, rice, and milk	Benchtop	1100–2300	SG-SNV-PLSR	R^2^p = 0.99; RMSEp = 0.023	[14]
	Supplements: whey, pea, glutamine, BCAA, and creatine	Melamine	Portable	900–1700	SNV-PLS	R^2^p = 0.998; RMSEp = 0.098	[67]
	Supplements: WPC	Maltodextrin, milk, and whey protein concentrate	Benchtop	1000–2500	SNV-PLSR	R^2^p = 0.977–0.995; RMSEp = 2.473–5.343	[80]
	Protein (whey, beef, and pea)	Melamine, urea, glycine, and taurine	Benchtop	1100–2200	SG-SNV-PLSR	R^2^cv = 0.95 ± 1.0; RMSEcv = 0.18–0.68	[68]
	High-quality commercial milk powder	Low-quality milk	Benchtop	1100–2498	SNV-NDF-kNN	Accuracy = 97.4%	[38]
	Infant formula milk powder	Melamine	Portable	980–1621	SG-VN-EMSC-PCA-LR	Accuracy = 100%	[49]
	Skimmed milk powder (SMP)	Melamine and Urea	Benchtop	850–2500	SG-EMSC-iPLS-PLSR	R^2^p = 1.0; RMSEp = 0.0016	[78]
Powdered fruits and their derivatives	Almond (*Prunus dulcis*)	Bitter almond (*Prunus amygdalus* var. amara) extract	Portable	740–1070	SG-SD-SNV-PLSR	R^2^p = 0.93; RMSEp = 0.079	[63]
	Almond (*Prunus dulcis*)	Cassava, oats, peanuts, and commercial flours	Benchtop	900–1700	SG-FD-OCPLS	Accuracy = 98.5%; Especificidad = 98.3%	[70]
	Melon seeds (*Cucumeropsis mannii*)	Corn, cassava, and soy	Portable	900–1700	SG-LDA	Accuracy = 99.05%	[64]
	Coconut milk	Corn and cassava	Benchtop	908–1676	SNV-GoogleNet/ResNet	R^2^p = 0.999; RMSEp < 0.0046	[84]
	Grape seed extract	Pine bark extract (PBE) and green tea extract (GTE)	Benchtop	400–2500	SG-MSC-PLSR	R^2^p = 0.993; RMSEp = 0.02	[73]
	Baobab	Rice, wheat, and corn	Portable	900–1700	SG-MC-PLSR	R^2^p = 0.98; RMSEp = 0.0274	[72]
	Dehydrated coconut powder (DCP)	Coconut milk	Portable	400–2400	Raw-PLSR	R^2^p = 0.973; SEP = 9.681	[106]
	GBF (GBF)	Wheat	Benchtop	400–2500	SD-SG-DT-PLS	R^2^p = 0.979; RMSEp = 0.0243	[90]
Cocoa and its powdered derivatives	Cocoa powder	Carob, cocoa husk, foxtail millet, soybean, and wheat	Benchtop	400–2500	Boruta-PLSR	R^2^p = 1.0; RMSEp < 0.0001	[42]
	Cocoa husk powder	Leaves, pods, stem fragments, and cocoa nibs	Portable	900–1700	SD-VIP-PLSR	R^2^p = 0.99; RMSEp < 0.0074	[43]
	Cocoa powder	Cocoa husk	Portable	900–1700	SG-FD-TD-EMCVS-PLSR	R^2^p = 0.939; RMSEp = 0.0069	[44]
Tubers	Maca (*Lepidium meyenii*): red, black, and yellow	Soy and corn products	Portable	900–1700	SG-PLSR	R^2^cv = 0.952; RMSEcv = 0.068	[45]
	Maca (*Lepidium meyenii*)	Rice and rice bran	Portable	900–1700	MSC-MC-SD-VIP-PLS-DA	Sensibilidad = 1.0; Especificidad = 1.0	[46]
	Maca (*Lepidium meyenii* Walp.)	Turnip and radish	Benchtop	400–2500	SD-MSC-siPLS	R = 0.977; RMSEp = 0.0579	[91]
Coffee and tea powder	Coffee var. Caturra	Toasted soybean, barley, chicory, and corn	Portable	900–1700	IWO-SVM	Accuracy = 92.25%; Especificidad = 99.42%	[23]
	Green tea	Sugar, rice	Portable	900–1700	SNV-IRIV-SVR	R^2^p = 0.998; RMSEp = 0.67	[85]
Legumes	Chickpeas and other legumes	Pea (*Pisum sativum* L.) and grass pea (*Lathyrus sativus* L.)	Benchtop	400–2498	SNV-DT-FD-MPLSR	R^2^c = 0.99; SEC < 0.905%	[86]
	Chickpea	Pea (Pisum sativum L.) and grass pea (*Lathyrus sativus* L.)	Benchtop	400–2498	SNV-DT-FD-MPLSR	R^2^c = 0.99; SEC < 1.092	[87]
Others	Insect protein	Proteins from fly (BSFL), cricket (*A. domesticus*), and mealworm (*T. molitor*)	Benchtop	800–2500	PLS	Q^2^ = 0.991–0.997; RMSEcv = 10.8–17.1	[95]
	Shrimp (*Caridea* sp.)	Immature shrimp and shrimp heads	Portable	900–1700	SG-MSC-PLSR	R^2^cv = 0.823; RPD = 2.99	[92]

Most studies using NIR spectroscopy for the detection of adulteration have focused on plant-based powdered foods, with an emphasis on matrices such as spices and seasonings, cereals and pseudocereals, dairy products and protein supplements, and fruit derivatives, tubers, and legumes. The main cases reported in the recent scientific literature are presented and analyzed below.

### 6.1. Powdered Spices and Seasonings

Powdered spices and seasonings are highly susceptible to adulteration because of their high commercial value, easily replicable color and texture, and global distribution. Turmeric (*Curcuma longa*) is one of the products most frequently studied in this context. Several studies have evaluated its adulteration with cereals, starches, and unauthorized dyes, obtaining highly satisfactory results through NIR spectroscopy combined with advanced algorithms. For example, the use of an SD-DT-SNV-PCA-CNN-1D model enabled the detection of adulterants such as corn, rice, and wheat with an R^2^p = 0.848 [39], whereas a SNV-PCA-RFR-based approach achieved an R^2^p = 0.999 for commercial starches [71]. In the case of adulteration with Sudan I, a common carcinogenic dye, the VIP-PLSR model showed excellent performance (R^2^p = 0.979; RMSEp = 0.0093) [65]. Similarly, Amsaraj et al. [81] reported an SNV-RCGA-XGBoost model with R^2^p = 0.999 and RMSEp = 0.0096 for the identification of multiple chemical and plant-based adulterants. Other approaches, such as SIMCA [62] and PCA-SVM [107], also demonstrated effectiveness in detecting spent or illegally pigmented turmeric, with accuracy levels exceeding 90%.

Adulteration with nutshells, flours, and hazelnuts has been detected in cinnamon. Cruz Tirado et al. [13] used a hierarchical PLS-DA model that achieved sensitivities of 0.8–0.9. Coqueiro et al. [75] achieved an R^2^p = 0.994 (RMSEp = 0.031) for the detection of coffee and corn bran, while a CNN model obtained 92.8% accuracy for hazelnut adulteration [97].

Powdered ginger, particularly its adulteration with beans and corn, has also been studied. Models such as MSC-PLS [40] and SG-SNV-Rfrog-PLSR [66] reported high R^2^ values (≥0.95) and low RMSEs, confirming their applicability to complex matrices. In paprika, the SG-PLSR and SNV-FD-PLSR combinations enabled high-precision detection of adulterants such as seeds, gums, and colorants [76,82]. Essuman et al. [98] applied a PLS-DA model to adulterate chili samples with avocado seeds and kola nuts, achieving 91.25% accuracy.

Adulterants such as nutshells, starches, and flours have also been evaluated in cumin and black pepper. In cumin, Cruz-Tirado et al. [93] developed an SG-SNV-PLSR model with RPD values between 3.61 and 4.39 and minimal prediction errors (RMSEp ≤ 0.006), while Lima et al. [109] reported an autoscaling-PLSR model for cumin and black pepper with an R^2^ = 0.95, confirming its ability to discriminate even in complex mixtures.

The reviewed studies demonstrate that NIR spectroscopy, combined with advanced chemometric models, such as PLSR, CNN, SVM, and XGBoost, enables effective and nondestructive detection of adulteration in powdered spices and seasonings.

### 6.2. Powdered Cereals and Pseudocereals

Powdered cereals and pseudocereals—such as buckwheat, rice, teff, and quinoa—are frequently adulterated with flours of lower nutritional value, including common wheat, cassava, oats, and corn. NIR spectroscopy coupled with advanced chemometric models has proven to be highly effective for detecting these adulterants in complex matrices.

In Tartary buckwheat (*Fagopyrum tataricum*), Yu et al. [24] employed an Autoscales-CARS-SVM model that achieved perfect classification (accuracy = 100%; F1-score = 1.0) when detecting whole wheat, oats, soy, barley, and sorghum mixtures. In addition, Chai et al. [83] applied an SNV-DT-CARS-PSO-SVR approach to distinguish Tartary buckwheat from common buckwheat, obtaining outstanding accuracy (R^2^p = 0.99; RMSEp = 0.0002). Unuvar et al. [110] used a Baseline-PLSR model for durum wheat, reaching an R^2^p of 0.867, demonstrating its utility in simpler adulteration scenarios.

Various strategies have been employed to detect adulteration in rice, both among varieties and with external matrices. Chen et al. [100] applied a BPNN model to differentiate premium rice varieties (Wuchang and Thai fragrant) adulterated with lower-value types, achieving R^2^p = 0.973 and RMSEp = 0.071. Rahmawati et al. [77] evaluated adulterated brown rice using SG-PLSR, reaching R^2^p = 0.96 and RMSEp = 0.004. Similarly, Teye et al. [105] implemented a Si-PLS model to identify substitutions between Jasmine 85 and Agra varieties, achieving R^2^p = 0.936 and RMSEp = 0.156.

Casarin et al. [22] also studied the pseudocereal teff (*Eragrostis tef*), who developed an MSC-SD-PLSR model to detect adulteration with rice, oats, and wheat, achieving R^2^p = 0.974 and RMSEp = 0.07. For quinoa (*Chenopodium quinoa* Willd), Wang et al. [102] reported exceptional performance using VIP-PLSR, with R^2^p = 0.98 and RMSEp = 0.0002, when detecting adulteration with wheat, rice, corn, cassava, and buckwheat.

These studies highlight the ability of NIR spectroscopy to discriminate between spectrally similar matrices and the critical role of proper preprocessing, variable selection, and optimal model choice in achieving high sensitivity and generalization—even in scenarios involving multiple adulterants at low concentrations.

### 6.3. Powdered Dairy Products and Supplements

Powdered dairy products and protein supplements represent a high-risk category in terms of adulteration due to their high commercial value and the ease with which they can be altered by adding nitrogenous substances such as melamine, urea, glycine, or maltodextrin. NIR spectroscopy, combined with both classical and advanced chemometric algorithms, has shown remarkable effectiveness in detecting such adulterations with high precision and without requiring destructive sample preparation.

Song et al. [14] applied an SG-SNV-PLSR model to detect mixtures with maltodextrin, milk, and wheat in flavored whey proteins, achieving an R^2^p = 0.99 and a very low RMSEp of 0.023. Similar results were obtained from Shutevska et al. [67], who used SNV-PLS to identify melamine in whey and pea protein supplements, achieving R^2^p = 0.998. Moghaddam et al. [80], for their part, evaluated adulterations involving multiple matrices and reported robust performance with R^2^p values ranging from 0.977 to 0.995, though with a greater spread in prediction errors (RMSEp = 2.473–5.343). However, the R2p values were not significant.

Lukacs et al. [68] extended the analysis to more complex mixtures of animal and plant-based proteins (whey, beef, and pea) adulterated with melamine, urea, glycine, and taurine. Their models, validated through cross-validation (R^2^cv = 0.95 ± 1.0; RMSEcv = 0.18–0.68), demonstrated the approach’s robustness. In commercial dairy products, Yuan et al. [38] applied an SNV-NDF-kNN model to discriminate high-quality milk from lower-purity versions, achieving an accuracy of 97.4%.

Particular attention must be paid to the detection of adulteration in infant formulas, where health risks are especially high. Ting et al. [49] used a multistage model (SGauss-VN-EMSC-PCA-LR) to detect melamine in infant milk powder, achieving 100% accuracy. Similarly, Ejeahalaka et al. [78] reported outstanding results for skimmed milk adulterated with melamine and urea, using an SG-EMSC-iPLS-PLSR model that achieved R^2^p = 1.0 and RMSEp = 0.0016.

These results confirm that NIR spectroscopy-based models are highly effective for detecting adulterants in powdered dairy products and protein supplements, even at low concentrations. Furthermore, they constitute key tools for rapid and nondestructive monitoring in regulatory and commercial contexts.

### 6.4. Plant-Based Products and Nuts

Powders derived from fruits and nuts, such as almond, melon, baobab, dehydrated coconut, coconut milk, and green banana, are frequently adulterated with low-cost ingredients, such as cassava, corn, rice, oats, or even flours from other plant sources. Adulteration compromises the authenticity, nutritional value, and functionality of these products, especially in contexts where they are marketed as functional foods or supplements.

In sweet almond (*Prunus dulcis*), Giussani et al. [63] used an SG-SD-SNV-PLSR model to detect substitution with bitter almond, achieving an R^2^p of 0.93 and an RMSEp of 0.079. Netto et al. [70] used SG-FD-OCPLS to identify mixtures with cassava, oat, and peanut flours, and other sources, reaching an accuracy of 98.5% and specificity of 98.3%. Zaukuu et al. [64] reported a precision of 99.05% using SG-LDA on melon seed samples adulterated with corn, cassava, and soy.

Coconut milk, a matrix of growing interest due to its use in vegan and functional foods, was evaluated by Sitorus et al. [84], who employed convolutional neural networks (GoogleNet/ResNet) with SNV preprocessing, obtaining outstanding results (R^2^p = 0.999; RMSEp < 0.0046). In grape seed extracts, Lukacs et al. [73] detected adulteration with compounds such as pine bark or green tea extracts using SG-MSC-PLSR, achieving R^2^p = 0.993.

Baobab fruit, which is highly valued for its antioxidant content, was analyzed by Yegon et al. [72], who applied SG-MC-PLSR to detect mixtures with rice, wheat, and corn, obtaining an R^2^p = 0.98. In the case of dehydrated coconut, Pandiselvam et al. [106] used a Raw-PLSR model to identify substitution with coconut milk, with results of R^2^p = 0.973 and SEP = 9.681. Finally, Ndlovu et al. [94] used an SD-SG-DT-PLS model in adulterated green banana with wheat, achieving R^2^p = 0.979 and RMSEp = 0.0243.

These studies highlight the utility of NIR spectroscopy combined with supervised models, such as PLSR, OCPLS, LDA, and deep neural networks, for the detection of multicomponent adulteration. The combination of appropriate spectral preprocessing and advanced algorithms has enabled the development of robust models, even in the presence of multiple adulterants and batch-to-batch variability, thereby consolidating this technique as an effective and nondestructive tool for authenticating powdered plant-derived products.

### 6.5. Cocoa, Coffee, and Derivatives

Cocoa powder and its derivatives are products of high sensory and economic value, making them particularly vulnerable to adulteration with low-cost inputs such as carob, cocoa husk, roasted cereals, soy, and foxtail millet. Owing to their visual similarity, these substances can go unnoticed through conventional inspection, highlighting the need for advanced spectroscopic methods for detection.

Millatina et al. [42] used a PLSR-based model over a wide spectral range (400–2500 nm) to detect multiple mixtures in cocoa powder, achieving outstanding performance (R^2^p = 1.0; RMSEp < 0.0001), demonstrating the ability of the technique to discriminate highly complex adulterations. In studies focused on cocoa husk powder, Oliveira et al. [43] applied SD-VIP-PLSR and SG-FD-TD-EMCVS-PLSR models, achieving coefficients of determination of 0.99 and 0.939, respectively, with very low prediction errors (RMSEp < 0.0074 and 0.0069), even when distinguishing between plant fragments such as leaves, stems, and nibs.

These results confirm that NIR spectroscopy, combined with variable selection algorithms and feature engineering techniques such as Boruta, EMCVS, and VIP, offers an effective, nondestructive, and sensitive solution for the authentication of cocoa powder. Its ability to detect fraud even when adulterants have physical or chemical similarity to the original matrix underscores its applicability in high-fidelity monitoring supply chains.

### 6.6. Tubers, and Other Powdered Foods

In recent years, NIR spectroscopy has been successfully applied to non-conventional matrices such as Andean tubers (e.g., maca), legumes (chickpea, pea), and alternative proteins such as insects or shrimp, demonstrating its versatility in the quality control of emerging or functional foods.

Zaukuu et al. [45] used an SG-PLSR model to detect adulteration with soy and corn in red, black, and yellow cultivars of maca (*Lepidium meyenii*), achieving remarkable accuracy (R^2^cv = 0.952; RMSEcv = 0.068). De Carvalho Rodrigues et al. [46] used a combination of spectral preprocessing and the MSC-MC-SD-VIP-PLS-DA model to distinguish between maca adulterated with rice and rice bran, achieving 100% sensitivity and specificity. Zeng et al. [91] also reported good results (R = 0.977; RMSEp = 0.0579) when using siPLS to discriminate mixtures with turnip and radish.

Zhang et al. [23] developed an IWO-SVM model to detect adulteration of Caturra coffee with roasted cereals and soy, reaching 92.25% accuracy and 99.42% specificity in coffee and tea-derived products. Similarly, Li et al. [85] evaluated green tea samples adulterated with sugar and rice using SVR combined with variable selection (IRIV), achieving R^2^p = 0.998 and RMSEp = 0.67. Legumes have also been studied. Bala et al. [86,87] used SNV-DT-FD-MPLSR models on chickpea and pea samples adulterated with *Lathyrus sativus* or corn, obtaining R^2^c = 0.99 in both cases, with SEC below 1.1%.

Finally, promising results have been achieved in matrices of animal and alternative origin such as insect proteins (BSFL, crickets, mealworms) or shrimp. Ni et al. [95] applied PLS to samples adulterated with various insect species, obtaining Q^2^ = 0.991–0.997 and RMSEcv between 10.8 and 17.1. Similarly, Zaukuu et al. [92] used SG-MSC-PLSR to detect the presence of heads and immature shrimp in shrimp powder, achieving R^2^cv = 0.823 and an RPD = 2.99, which are acceptable metrics for discriminative applications.

These studies demonstrate that NIR spectroscopy, complemented by robust algorithms and appropriate preprocessing techniques, enables authenticity analysis across a wide range of non-traditional matrices. This significantly expands the technology’s reach toward functional ingredients, alternative proteins, and innovative foods with growing global market presence.

## 7. Current Challenges and Future Trends

**Figure 3:**
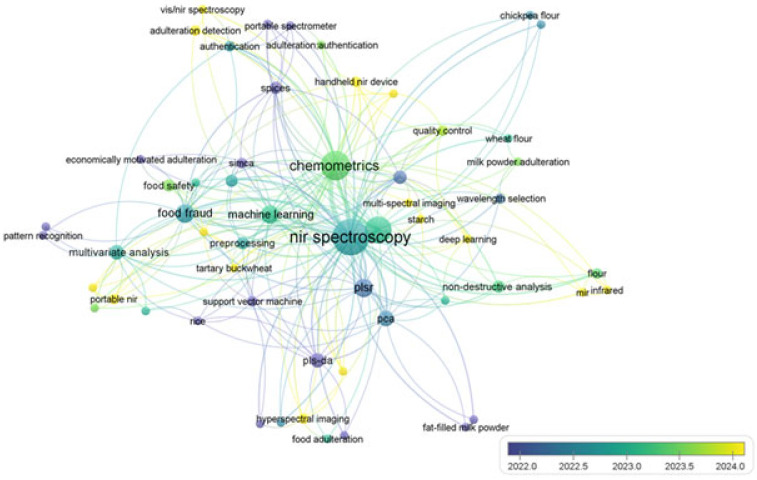
Co-occurrence Map of Author Keywords in Food Adulteration Detection Using NIR Spectroscopy (2020–2025) Studies. Figure was generated in VOSviewer [1.6.20].

The thematic evolution of recent literature on the detection of adulterants in powdered foods using NIR spectroscopy reflects a substantial shift in research priorities (See Figure 3). Trend analysis (2020–2025) shows a transition from classical statistical approaches toward more integrated models combining artificial intelligence and digital tools.

### 7.1. Evolution of Thematic Trends

During the initial years of the analyzed period, the most frequent terms were associated with traditional chemometric models, such as PLSR, PCA, and PLS-DA, all with publication peaks between 2021 and 2023. These algorithms served as the analytical foundation for classifying and quantifying adulterants in coffee, turmeric, milk powder, and cereals.

From 2023 onwards, and especially in 2024, the most recurrent terms shifted toward practical and operational applications, highlighting “nir spectroscopy” (29 mentions), “adulteration” (23 mentions), and “food fraud” (11 mentions), indicating a growing focus on real-time authenticity validation and risk assessment related to food fraud.

Simultaneously, terms such as “chemometric” (21 mentions) remain central to methodology, while “curcumin” reflects the interest in specific, high-value plant-based matrices. The notable emergence of “machine learning” in 2024 marks a significant methodological inflection, anticipating broader adoption of more complex algorithms capable of self-calibration and adaptation to new forms of adulteration.

This shift in trends demonstrates an evolution from methodological approaches toward practical applications in the detection of food fraud. Although interest in machine learning and real-time validation is increasing, greater effort is still needed to transfer these advances into industrial environments. Future studies must incorporate field validations and regulatory considerations to achieve a tangible impact on food safety.

### 7.2. Emerging Topics and Gaps

Trend analysis reveals several emerging areas of particular relevance. Among them, the sustained growth of supervised ML models and deep neural networks stands out, both of which have demonstrated high predictive capacity in complex matrices [63]. Likewise, notable progress has been made in studies focused on real-time authentication using portable NIR spectroscopy integrated into mobile platforms, opening new possibilities for rapid and decentralized inspection [41]. Another expanding line of research involves the analysis of food products with high economic vulnerability and added value, such as spices, supplements, and gluten-free flours, which have become frequent targets of fraud.

However, significant gaps exist. Key concepts, such as blockchain, calibration transfer, Internet of Things (IoT), and spectral digital twins, which are essential for advancing automated chemical traceability and interoperability between devices, still lack a consolidated presence in the literature. Although spectroscopic tools have evolved technically, their integration into intelligent digital environments is still in an early phase. Bridging this gap will be crucial for implementing robust, scalable, and adaptable authentication solutions that meet the demands of the modern food industry.

### 7.3. Future Projections

Based on the observed evolution, future NIR spectroscopy research applied to food adulteration detection is expected to move toward greater technological and operational integration. A stronger link between NIR and decentralized digital platforms, such as the IoT and blockchain, is anticipated. This would enable the implementation of more secure, automated, and real-time chemical traceability systems. Additionally, data fusion from multiple sources—such as NIR spectra, RGB images, and sensory measurements—will become increasingly important for enhancing the accuracy and robustness of authentication models. The development of self-adjusting and transferable models capable of maintaining performance across different devices, laboratories, or manufacturers is another key focus. Finally, the progressive incorporation of NIR spectroscopy into predictive regulatory control systems is foreseen, enabling continuous, remote verification of food authenticity without the need for specialized laboratories.

## 8. Conclusions

Near-infrared (NIR) spectroscopy, combined with advanced chemometric techniques, is a powerful, nondestructive, and versatile alternative to traditional methods for detecting adulterants in powdered foods. Compared with conventional techniques, such as chromatography or wet chemistry—which are often labor-intensive, destructive, and require elaborate sample preparation—NIR enables rapid, real-time, and cost-effective screening with minimal handling. Its ability to analyze complex food matrices without altering their physical integrity makes it particularly valuable for quality control, authentication, and fraud prevention across the entire supply chain.

The literature reviewed from 2020 to 2025 demonstrates that the integration of NIR with machine learning algorithms (e.g., PLSR, SVR, SVM, XGBoost, and CNN), supported by effective spectral preprocessing (e.g., SNV, derivatives, and EMSC), has resulted in highly accurate models that frequently exceed 95% accuracy—even in cases involving multi-component or low-level adulteration. However, significant challenges persist. Equipment heterogeneity affects model transferability and reproducibility across platforms, including differences in resolution, detector sensitivity, and spectral range. Detecting subtle adulterations still requires careful model tuning, optimized preprocessing, and extensive validation. The lack of standardized methodologies and regulatory harmonization further constrains the broader implementation of NIR technologies, especially in small-scale industries and low-resource environments.

To address these limitations and fully unlock the potential of NIR spectroscopy, future development must focus on its integration with intelligent digital infrastructures. The convergence of NIR with IoT systems, cloud-based analytics, and blockchain-enabled traceability will allow for real-time, adaptive, and interconnected monitoring solutions. This transition from laboratory-bound analysis to portable, scalable, and connected platforms marks a technological advancement and a strategic imperative for ensuring transparency, authenticity, and safety in modern food systems. When appropriately deployed, NIR spectroscopy stands not merely a tool for detection—it becomes a cornerstone technology in the global fight against food fraud.

## Figures and Tables

**Table 1:** Spectral preprocessing techniques applied in NIR-based food authenticity studies on powdered products.

Technique	Main Purpose	Effect	Source
Savitzky–Golay (SG)	Smoothing of the high-frequency noise	Improves the signal-to-noise ratio and spectral stability	[14,23,41,43,45,49,63,64,66,68,70,72,73,74,75,76,77,78,79]
Standard Normal Variate (SNV)	Correction of the scattering variations	Enhances class separation and sensitivity and specificity	[38,39,63,66,67,68,71,73,80,81,82,83,84,85,86,87,88]
First Derivative (FD)	Highlight subtle changes and remove the baseline	Emphasizes minor compounds and requires additional smoothing (SG)	[41,43,70,86,87,89]
Second Derivative (SD)	Enhancing class discrimination	Improves class separation; requires additional SG	[22,39,43,46,63,90,91]
Multiplicative Scatter Correction (MSC)	Correction of the additive/multiplicative scatter	Improves model robustness and enhances PLSR performance	[22,40,46,73,91,92]
Detrending (DT)	Removing nonlinear baseline trends	Complements SNV and improves multi-class classification	[39,83,86,87,90]
Mean Centering (MC)	Standardize the spectral scale	Improves PCA discrimination and is useful with SD and MSC	[46,72]
Extended Multiplicative Signal Correction (EMSC)	Correcting complex systematic variations	Enhances discrimination and robustness of SIMCA/PLSR	[49,78]
Gaussian smoothing (GS)/Cut	Attenuate noise/limit useful range	Increases accuracy of models with restricted wavelength range	[49]
Min-Max Normalization	Scale of spectral data	Improves model fitting and reduces overfitting	[65]

**Table 2:** Feature selection techniques applied in NIR-based food authenticity studies on powdered products.

Technique	Principle	Advantages	Limitations	Source
Principal Component Analysis (PCA)	Orthogonal transformation to capture the maximum variance	Efficient dimensionality reduction	Does not identify specific variables but combines components	[39,41,49,65,66,69,70,71,75,80,86,95,97,98]
Competitive Adaptive Reweighted Sampling (CARS)	Adaptive selection based on the PLS regression weights	High accuracy in large spectral datasets	Dependence on stochastic parameters	[66,83,88,99,100]
Successive Projections Algorithm (SPA)	Orthogonal variable selection with minimal collinearity	Avoids redundancy and improves interpretability	Risk of excessively removing useful variables	[80,99,100,101]
Variable Importance in Projection (VIP)	Identifies each variable’s relative importance in PLS	Direct interpretability and low computational cost	Sensitivity to the number of PLS components	[22,46,65,102]
PLS beta coefficients	Key wavelengths are identified via absolute values of regression coefficients	Fast, easy to interpret, and useful for identifying spectral regions	May eliminate useful variables in complex data; being linear, it is best when combined with more robust methods	[65,76,85]
Random Frog (RFrog)	Stochastic sampling to explore the frequently selected variables	Robust exploration of the variable space	Sensitive parameters, no guaranteed optimum	[66,88]
Real Coded Genetic Algorithm (RCGA)	Evolutionary optimization with real-variable encoding	High capacity for nonlinear optimization	Iterative evaluation is computationally expensive	[81]
Invasive Weed Optimization (IWO)	Adaptive seed dispersion as evaluated by fitness	Escapes local optima through random dispersión	Sensitivity to parameter configuration (number of iterations, population size)	[23]
Binary Chimpanzee Optimization Algorithm (BChOA)	Chaotic cooperative hunting for global variable search	Diverse exploration using chaotic maps	Complex configuration dependent on chaotic parameters	[23]
Separation Degree Priority Combination (SDPC)	Supervised PCA with class separation maximization	Supervised separation of the latent classes	Reliable labels are required; limited commercial implementation	[38]
Wavelength Step-by-step Phase-out (WSP)	Weighted spectral grouping with informed projection	Suitable for dispersed adulterants	Dependent on the initial grouping	[38]
IRIV (Iteratively Retaining Informative Variables)	Iterative evaluation of variables’ statistical relevance	Fine filtering of the informative variables	High computational cost	[85]
Regression Coeficients (RC)	Hierarchical clustering and recursive cluster evaluation	Interactions captured in correlated spectral bands	Not optimal for isolated spectral effects	[69]

**Table 3:** Qualitative prediction models applied in NIR-based food authenticity studies on powdered products.

Model	Typical Application	Advantages	Limitations	Source
Partial Least Squares Discriminant Analysis (PLS-DA)	Binary/multiclass classification in linearly structured spectral matrices	Easy to interpret and suitable for linear spectra	Low performance on nonlinear data	[13,41,46,69,72,75,80,82,98,99,105,106]
Support Vector Machine (SVM)	Nonlinear and multiclass classification: useful for complex adulteration cases	High accuracy; effective on nonlinear data	Parameter tuning and appropriate normalization are required.	[23,42,66,69,85,91,98,99,105,107]
Linear Discriminant Analysis (LDA)	Separation of linearly separable classes; ideal for simple spectra	Fast and low computational demand	Inefficient for spectral nonlinearity	[45,64,66,68,69,92,105]
Random Forest (RF)	Robust classification of large spectral datasets	High performance with multiple variables	Complex to interpret and prone to overfitting	[42,69,71,81,105]
SIMCA (Soft Independent Modeling of Class Analogy)	One-class modeling for authentication with no known adulterant	Suitable for authentication without requiring a negative class	Limited to well-defined cases	[70,74,78,108,109]
Data-Driven SIMCA (DD-SIMCA)	Adaptive multiclass authentication without a negative reference	High sensitivity in scenarios with no negative class effects	Strong external validation strategy is required	[13,70,93]
K-Nearest Neighbors (kNN)	Simple classification based on the spectral distance	Intuitive; effective in small datasets	Sensitivity to noise and choice of k	[38,80]
Convolutional Neural Network-1D (CNN-1D)	Deep classification in large nonlinear matrixes	Capture complex nonlinear and structural relationships	Requires high computational power	[39,97]
XGBoost	Ensemble of sequential decision trees, each of which corrects previous errors	High predictive performance; transforms weak learners into strong learners; robustness to collinearity and overfitting	Extensive and computationally expensive hyperparameter optimization	[81]
One-Class Partial Least Squares (OCPLS)	One-class modeling to describe the target class’s spectral distribution	Maximizes the explained variance of the authentic class; correlates spectra with a fixed reference value (=1); no need for negative class information	Low sensitivity to low concentrations of adulterants (<5%)	[70]
OPLS-DA (Orthogonal Partial Least Squares-Discriminant Analysis)	Differentiating predictive from orthogonal (non-predictive) information	Accurately identifies relevant spectral variables; enhances model clarity and stability	Reduced sensitivity, limiting its ability to classify authentic/genuine samples correctly	[109]

**Table 4:** Quantitative prediction models applied in studies of the authenticity of powdered food using NIR spectroscopy.

Model	Typical Application	Advantages	Limitations	Source
Partial Least Squares Regression (PLSR)	Prediction of adulterant levels from the full spectrum	Robust collinearity; widely validated	Sensitivity to the number of latent components	[22,40,42,44,45,63,65,70,72,73,75,78,80,82,83,85,88,90,92,93,95,98,100,102,105,109,110]
Support Vector Regression (SVR)	Nonlinear regression in multicomponent matrixes	High accuracy in nonlinear spectra and flexible	Requires careful parameter tuning	[65,73,83,85,100]
Principal Component Regression (PCR)	Regression on the principal components for the collinear data	Reduces redundancy and is easy to interpret	Lower accuracy in the nonlinear spectra	[65,77,86,87]
Random Forest Regressor (RFR)	Robust estimation of high-variability data	Noise tolerance; no normal distribution required	Harder to interpret and prone to overfitting	[42,71,81,107]
Multilayer Perceptron Regressor (MLP)	Modeling complex nonlinear spectral relationships	Learns complex relations and adapts to multiple classes	High computational demand and risk of overfitting	[76,109]
iPLS (Interval Partial Least Squares)	Localized prediction using spectral segments	Focus on relevant intervals; improves signal-to-noise	Sensitive segmentation: risk of losing global information	[78,105]
Si-PLS (Synergy Interval Partial Least Squares)	Combined optimization of the spectral segments	Captures synergy between the relevant bands	Tuning optimal interval combinations is difficult	[91,105]
MPLSR (Regresión modificada por Mínimos Cuadrados Parciales)	Enhances classical PLSR with smoothing and robustness to noise and collinearity	High accuracy, low error, and high computational efficiency	--	[86,87]
DTR (Decision Tree Regression)	Hierarchical modeling of nonlinear decision-making	Intuitive, fast, and interpretable	Lower accuracy and decision fragmentation	[87]
Back Propagation Neural Network (BPNN)	Multivariate prediction via backpropagation	Flexible with many layers; suitable for large spectral data	Requires careful tuning and is prone to overfitting	[100]
Long Short-Term Memory (LSTM)	Temporal memory modeling in multiclass spectral data	Capture time-dependent spectral relations	Needs sequential data and extensive training	[76]
GBT (Gradient Boosted Tree)	Error boosting optimization	High accuracy; handles irrelevant variables	Computational intensive; hyperparameter tuning	[107]
kNNR (k-Nearest Neighbors Regression)	Estimation by spectral proximity	Useful for spectrally similar simples	Sensitive to outliers; dependent on the k value	[71]
XGBoost Regressor	Efficient tree-based ensemble model	High predictive power and scalable	Hard to optimize; limited interpretability	[81]
Linear Regression	Direct modeling of adulterant concentration	Simple, fast, and easy to understand	Limited to simple linear relationships	[107]
S-AlexNET	Extracts relevant spectral features automatically without manual variable engineering	High predictive ability, low overfitting, and spectral regions that are interpretable	High computational cost	[84]
Res-NET	Deep CNN with residual connections for SP learning	Automated feature extraction; high robustness and predictive capacity; interpretable	High computational cost	[84]
GoogleNET	Inception modules are used to capture spectral patterns at multiple scales	Automated feature extraction; robust against overfitting	High computational cost	[84]
LASSO	L1-regularized regression that automatically selects relevant variables while the predictive model is fitted	Controls overfitting, reduces dimensionality, and is efficient for collinear spectral data	--	[42]
Ridge	L2-regularized regression that shrinks the magnitudes of coefficients without eliminating them	Stable in collinear spectral matrices	--	[42]
ElasticNET	Combination of L1 (LASSO) and L2 (Ridge) penalties for simultaneous regularization and variable selection	Robust performance, dimensionality reduction, and computational efficiency	--	[42]
ETR (Extra Tree Regressor)	Ensemble model with increased randomness in the selection of node threshold	Noise-robust and capable of modeling nonlinear relationships	--	[71]

**Table 5:** Evaluation Metrics for the Qualitative Models.

Metric	Purpose	Application	Equation	Source
Accuracy	Measures the overall accuracy	For the balanced sets	$\frac{TP+TN}{TP+TN+FP+FN}$	[23,24,66]
Sensitivity/Recall	Detects positives	minimize the occurrence of false negatives	$\frac{TP}{TP+FN}$	[13,22,64]
Specificity	Detects negatives	minimize the occurrence of false positives	$\frac{TN}{TN+FP}$	[13,23,45]
Precision	Reliability of the positive predictions	Relevant when false positives are costly	$\frac{TP}{TP+FP}$	[37,45,69,80]
F1-Score	Balance between the precision and the recall	Suitable for imbalanced class scenarios	$2\times \frac{Precision\times Sensitivity}{Precision+Sensitivity}$	[37,69,81]

Note. *TP*, true positives are the number of actual positive samples that the model correctly classified as positive; *TN*, true negatives are the number of negative samples that the model correctly classified as negative; *FP*, false positives are the number of negative samples that the model incorrectly classified as positive; *FN*, false negatives are the number of positive samples that the model incorrectly classified as negative.

**Table 6:** Evaluation Metrics for the Quantitative Models.

Metric	Purpose	Application	Equation	Source
R^2^	Variance explained by the model	Evaluates the fit of predictions with reference values (adulteration levels)	$1-\frac{\sum {\left(y_{i}-{\hat{y}}_{i}\right)}^{2}}{\sum {\left(y_{i}-\bar{{\hat{y}}_{i}}\right)}^{2}}$	[22,63,84]
RMSE	The magnitude of error	Indicates the accuracy with which the model predicts the adulteration level	$\sqrt{\frac{\sum {\left(y_{i}-{\hat{y}}_{i}\right)}^{2}}{n}}$	[22,63,84]
Bias	Systematic trend	This allows us to determine whether the model tends to systematically overestimate or underestimate the adulterant.	$\frac{\sum \left(y_{i}-{\hat{y}}_{i}\right)}{n}$	[84]
SEP	Corrected prediction error	Eliminates the influence of bias in error calculation	$\sqrt{\frac{\sum {\left(y_{i}-{\hat{y}}_{i}-Bias\right)}^{2}}{n-1}}$	[37,40]
RPD	Robustness and practical utility of the proposed model	evaluating the practical capacity of the model	$RPD=\frac{SD}{RMSE}$	[45,63,84]
RER	Ratio/error	The relationship between the actual variability of the samples and the prediction error is indicated.	$\frac{\left(y_{max}-y_{min}\right)}{RMSE}$	[43,75]

Note. $y_{i}$ is the reference value of sample “i”; ${\hat{y}}_{i}$ is the value predicted by the model for sample “i”; ${\bar{y}}_{i}$ is the average value of actual/observed values $y_{i}$; n is the number of samples/observations; SD is the standard deviation; $y_{max}$ is the highest reference value; $y_{min}$ is the lowest reference value.

**Table 7:** Software packages for NIR chemometric analysis.

Software	Type	Main Capabilities	Advantages	Limitations	Source
MATLAB (R2020a–R2025a)	Commercial	Numerical programming environment with a specialized toolbox (PLS Toolbox) enabling preprocessing, feature selection, modeling (supervised, unsupervised, machine learning, deep learning), and validation.	Robust and flexible platform; research standard; direct integration with toolboxes; PLS Toolbox graphical interface facilitates use without programming; specialized technical support.	High cost (requires MATLAB license + toolbox license). Moderate to High Learning Curve	[112,113,114]
Unscrambler X10.2–X12.1 (Camo Analytics)	Commercial	Experimental design, principal component analysis, PLS, supervised and unsupervised classification.	Intuitive graphical interface; automatic reporting; widely used in industry	High cost and less flexibility for novel algorithms.	[115,116,117,118]
R (4.0.1–4.5.0)	Free/Open-source	A wide range of regression and classification algorithms, spectral preprocessing, validation, and visualization.	Free, highly reproducible, and flexible; large scientific community.	Requires programming knowledge. High learning curve	[119,120,121]
Python (3.8.0–3.13.0)	Free/Open-source	A wide range of regression and classification algorithms, spectral preprocessing, validation and visualization, and integration with spectroscopic data are all included.	Free, scalable, and strongly supports AI and deep learning.	Requires programming knowledge. High learning curve	[122,123,124,125,126]

**Table 8:** Adulterants in powdered foods: health and nutritional implications.

Food	Adulterant	Nutritional Impact	Health Risks	Source
Milk and supplements	Melamine, urea	Artificial increase in nitrogen	Kidney damage, fatal in infants	[108]
Sweet almond	Bitter almond	Increased toxic amygdalin levels	Cyanide toxicity	[63]
Turmeric	Sudan I (1-[(2,4-dimetilfenil)azo]-2-naftalenol); Metanil Yellow	Reduction of curcuminoids production	Potential cancer risk and hepatotoxicity	[65,74]
Black pepper	Papaya seed	Reduction in piperine	Possible toxicity	[109]
Cumin	Nut shells (e.g., walnut, pecan, and peanut)	Dilution of the bioactive compounds	Severe allergic reactions	[93]
Maca	Rice and rice bran	Protein reduction	Undesired metabolic effects	[46]
Coffee	Soy	Reduction in the levels of caffeine and polyphenols	Allergy to the soy components	[23]
Teff	Wheat	Reduction in protein and mineral content	Gluten allergy	[22]
Buckwheat	Wheat	Reduction in the amount of soluble fiber and phenolics	Gluten allergy	[99]
Wheat	Talc powder and benzoyl peroxide	Nutrient dilution, oxidizing effect of PBO	Long-term toxic and carcinogenic effects	[88]

**Table 9:** Differential Characteristics of the Portable and Benchtop NIR Devices.

Feature	Portable Devices	Benchtop Devices
Typical spectral range	900–1700 nm	900–2500 nm
Spectral Resolution	10–20 nm	≤2 nm
Spectral Dimension	10–100 bands	100–1000 bands
Optical Geometry	Diffuse reflectance	Transmittance, diffuse reflectance, and integrating sphere
Detector Type	Miniature InGaAs	High-sensitivity InGaAs
Light Source	LEDs or halogen lamps	Halogen lamps
Compatible Sample Formats	Powders on optical windows or plastic bags	Solids, liquids, and powders in the sample holders
Main Applications	Rapid detection of in situ adulteration	Laboratory quality control and generation of reference spectra
Key Advantages	Portability, speed, and ease of use	High precision, reproducibility, and advanced multivariate analysis
Limitations	Lower resolution, environmental interferences, and limited multiclass detection	Expensive, non-portable, and slower sampling speed

Note. Information extracted from the scientific literature reviewed in this study, including references [13,14,22,24,39,41,43,46,63,65,66,67,69,70,75,76,83,88,92,102,109,128].

## Data Availability

No new data were created or analyzed in this study. Data sharing is not applicable to this article.

## Abbreviations

B	
BPNN	Back Propagation Neural Network
BSFL	Black Soldier Fly Larvae
C	
CARS	Competitive Adaptive Reweighted Sampling
C-H	Carbon–Hydrogen bond
CNN	Convolutional Neural Network
CNN-1D	One-Dimensional Convolutional Neural Network
CV	Cross-Validated
D	
DD-SIMCA	Data-Driven Soft Independent Modeling of Class Analogy
DT-PLS	Definition not provided
DT	Detrending
DTC	Decision Tree Classifier
DTR	Decision Tree Regressor
DNA	Deoxyribonucleic acid
E	
ELM	Extreme Learning Machine
EMCVS	Extended MC-based Variable Selection
EMSC	Extended Multiplicative Signal Correction
ETC	Extremely Randomized Trees Classifier
ETR	Extra Trees Regressor
F	
FAO	Food and Agriculture Organization
FD	First Derivative
FT-IR	Fourier Transform Infrared Spectroscopy
FT-NIR	Fourier Transform Near Infrared Spectroscopy
G	
GTE	Green Tea Extract
H	
HPLC	High-Performance Liquid Chromatography
I	
IARC	International Agency for Research on Cancer
IoT	Internet of Things
IRIV	Iteratively Retained Informative Variables
IWO	Invasive Weed Optimization
K	
KNC	K-Nearest Centroid
KNN	K-Nearest Neighbors
KNR	K-Nearest Neighbors Regression
KS	Kennard-Stone Sampling
KSPXY	kernel distance-based sample set partition based on joint x-y distances
L	
LASSO	Least Absolute Shrinkage and Selection Operator
LDA	Linear Discriminant Analysis
M	
MAE	Mean Absolute Error
MC	Mean Centering
MIR	Mid-Infrared Spectroscopy
MLP	Multilayer Perceptron
MLR	Multiple Linear Regression
MPLSR	Modified Partial Least Squares Regression
MSC	Multiplicative Scatter Correction
N	
N-H	Nitrogen–hydrogen bond
NIR	Near-Infrared Spectroscopy
NN	Neural Network
O	
OCPLS	One-Class Partial Least Squares
O-H	Oxygen–hydrogen bond
OPLS-DA	Orthogonal Partial Least Squares Discriminant Analysis
P	
PCA	Principal Component Analysis
PCR	Principal Component Regression
PLS-DA	Partial Least Squares Discriminant Analysis
PLS	Partial Least Squares
PSO	Particle Swarm Optimization
R	
RARP	Recognition-Accuracy Rate in Prediction
RARV	Recognition-Accuracy Rate in Validation
RCGA	Real-Coded Genetic Algorithm
RER	Range Error Ratio
RFC	Random Forest Classifier
RFR	Random Forest Regression
RGB	Red-Green-Blue
RMSE	Root Mean Square Error
RMSECV	Root Mean Square Error of Cross-Validation
RMSEP	Root Mean Square Error of Prediction
ROBPCA	Robust Principal Component Analysis
ROC	Receiver Operating Characteristic
RP	Reflectance Profile
RPD	Residual Predictive Deviation
S	
SD	Second Derivative
SDG	Sustainable Development Goals
SDPC	Successive Derivative Preprocessing and Classification
SEC	Standard Error of Calibration
SEP	Standard Error of Prediction
SG	Savitzky–Golay
SIMCA	Soft Independent Modeling of Class Analogy
SNR	Signal-to-Noise Ratio
SNV	Standard Normal Variate
SPA	Successive Projections Algorithm
SPXY	Sample Set Partitioning based on joint X–Y distances
SVM	Support Vector Machine
SVR	Support Vector Regression
T	
TD	Third Derivative
V	
VIP	Variable Importance in Projection
VIS-NIR	Visible and Near-Infrared Spectroscopy
VN	Vector Normalization
W	
WSP	Wavelength Step-by-step Phase-out
X	
XDS	Xenon Discharge Source
